# A network of basolateral amygdala projection neurons contributes to stress-induced activation of the hypothalamic-pituitary-adrenal axis

**DOI:** 10.1126/sciadv.adv3737

**Published:** 2025-11-07

**Authors:** Robert J. Aukema, Gavin N. Petrie, Benjamin K. Lau, Lauren T. Seabrook, Samantha L. Baglot, John P. Christianson, Jaideep S. Bains, Maria Morena, Stephanie L. Borgland, Matthew N. Hill

**Affiliations:** ^1^ Neuroscience Graduate Program, University of Calgary, Calgary, Canada.; ^2^ Hotchkiss Brain Institute, Cumming School of Medicine, University of Calgary, Calgary, Canada.; ^3^ Mathison Centre for Mental Health, Cumming School of Medicine, University of Calgary, Calgary, Canada.; ^4^ Department of Physiology and Pharmacology, Hotchkiss Brain Institute, University of Calgary, Calgary, Canada.; ^5^ Department of Psychology & Neuroscience, Boston College, Chestnut Hill, MA, USA.; ^6^ Department of Physiology and Pharmacology, Sapienza University of Rome, Rome, Italy.; ^7^ Neuropsychopharmacology Unit, European Center for Brain Research, Rome, Italy.; ^8^ Departments of Cell Biology & Anatomy and Psychiatry, Cumming School of Medicine, University of Calgary, Calgary, Canada.

## Abstract

The basolateral amygdala (BLA) is reliably activated by psychological stress in both humans and rodents and influences diverse behavioral and physiological processes involved in stress adaptation. However, functional organization of distinct BLA circuits and their contribution to stress-induced activation of the neuroendocrine response is unclear. We establish four major findings in adult male rats: (i) BLA projection neurons are necessary and sufficient for stress-induced neuroendocrine activation; (ii) projection populations have a heterogeneous spatial distribution across the BLA; (iii) diverse BLA populations targeting the prelimbic cortex, nucleus accumbens, bed nucleus of stria terminalis, central amygdala, lateral hypothalamus, and ventral hippocampus are activated by acute stress, with the location of activated populations biased toward the medial basal amygdala; and (iv) inhibition of singular projections does not recapitulate global inhibition of BLA projection neurons. Together, this suggests that a network of BLA projection populations is broadly activated by acute stress and collectively contribute to neuroendocrine regulation.

## Teaser

A network of basolateral amygdala neurons, but not singular projections, contributes to the neuroendocrine response to stress.

## INTRODUCTION

Exposure to aversive, stressful stimuli leads to a widely encompassing biological response that prepares an organism for potential harm through various behavioral, physiological, hormonal, and autonomic responses (*1*–*3*). Regardless of the nature or temporal dynamics of psychological stressors, all stressors lead to activation of the paraventricular nucleus of the hypothalamus (PVN), the main driver of the hypothalamic-pituitary-adrenal (HPA) axis, and subsequent release of glucocorticoid hormones [cortisol in humans; corticosterone (CORT) in rodents] (*1*, *4*). The magnitude of HPA axis activation, however, is often reflective of stressor intensity (*5*, *6*). Although adaptive in the short term, prolonged or unnecessary activation of the stress response can lead to a wide range of health concerns (*7*). As such, an essential question is how the brain appropriately coordinates and regulates a response to psychological stressors.

A diverse network of brain regions regulates HPA axis activation during exposure to psychological stress, including the bed nucleus of the stria terminalis (BST), medial prefrontal cortex, ventral hippocampus (VH), and lateral septum (*8*–*12*); however, these regions largely act to constrain HPA axis activation or promote its termination following cessation of stress. On the other hand, we have a limited understanding of neural circuits that contribute to the activation of the HPA axis in response to acute psychological stress.

Anatomically, the BLA is well situated as a stress-regulatory region. It receives input encoding all sensory modalities (*13*, *14*), which can be integrated with cognitive information encoding memory and motivational drive, to subsequently modulate various physiological and behavioral responses through widespread projections to effector regions via calcium- and calmodulin-dependent protein kinase II–positive (CaMKII+) glutamatergic projection neurons (*15*). The BLA is reliably activated by psychological stress in both humans and rodents (*16*–*19*), and amygdala hyperactivity is strongly implicated in human stress disorders (*20*–*22*). Furthermore, preclinical research implicates the BLA in many stress-related processes, including avoidance behavior (*23*, *24*), autonomic activation (*25*), and memory (*26*). Collectively, this has established the BLA as a critical stress “hub” (*17*, *27*).

The BLA is a complex structure, however, and is also activated by—and drives—behavioral responses supporting appetitive and “positive” stimuli such as reward (*28*–*31*) and safety (*32*–*34*). Emerging literature suggests that distinct subpopulations of projection neurons in the BLA encode and drive opposing functions, particularly in the context of valence-encoding (*28*, *35*, *36*), fear expression (*32*), and avoidance behavior (*23*, *37*). These subpopulations are largely defined by circuit (*36*, *38*–*43*) or molecular identity (*33*, *35*, *44*–*46*), for which there are diverse expression gradients across the BLA (*28*, *35*, *47*–*51*) and which mutually interact via inhibitory interneurons (*28*, *36*). Although BLA neurons with opposing function are intermingled throughout the BLA (*28*, *31*, *36*, *52*), aversive stimuli appear to drive a bias in activation toward the anteromedial basal amygdala (*19*, *35*, *50*, *53*, *54*), suggesting that this is a particular important subregion during stress.

Despite a prominent role in many behavioral aspects of stress, there is an unexpectedly limited understanding of the role of the BLA in the regulation of the HPA axis (*27*). Although broad lesions of the amygdala inhibit stress-induced HPA axis activation (*55*, *56*), intra-BLA pharmacological manipulations targeting discrete neuromodulatory systems have divergent effects, with some altering stress-induced CORT (*57*–*60*) and others having no effect (*55*, *61*–*63*). Likewise, experiments in several animal species and human patients with epilepsy suggest that the effect of electrical or chemical stimulation of the BLA on the CORT response is highly variable and is dependent on the precise location of the BLA targeted (*64*–*71*).

We have previously characterized activation gradients in response to a wide range of different psychological stressors, identifying a bias in stress-induced activation toward the medial basal amygdala (mBA) (*19*, *50*). Collectively, this supports a hypothesis that distinct BLA anatomical subregions may be involved in initiation of the HPA axis response to psychological stress; however, to date, the projection identity and specific role of stress-sensitive BLA subregions in activation of the HPA axis remain unknown. This work addresses three research questions: (i) How are projection neuron populations organized within the BLA and specifically within the mBA? (ii) Which BLA projection neurons are activated by acute psychological stress? (iii) Do individual projection neuron populations contribute to activation of the HPA response to stress?

## RESULTS

### Inhibition of BLA projection neurons attenuates a stress-induced CORT response

**Fig. 1.**
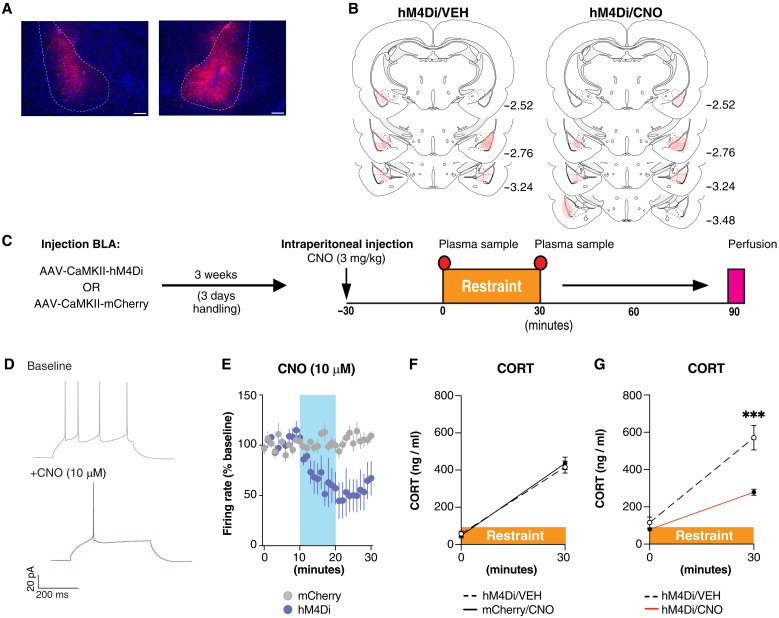
Inhibition of BLA projection neurons attenuates a stress-induced CORT response. (**A**) Representative image of hM4Di-mCherry expression. Dashed lines delineate BLA. Scale bars, 250 μm. (**B**) Representative images of hM4Di-mCherry expression in the BLA. Red indicates the area of maximal expression overlaid among all animals. *n* = 6 to 9 animals per group. (**C**) Overview of experimental procedures. (**D**) Representative traces of evoked action potentials before (baseline) and during the application of CNO (+10 μM). (**E**) Time plot of evoked firing before, during, and after the application of CNO (blue band). *N*/*n* = 8/3 (mCherry) and 6/4 (hM4Di). (**F**) Mean plasma CORT levels in hM4Di+ or mCherry+ animals at stress onset (0 min) and stress termination (30 min) 30 min following the intraperitoneal injection of VEH or CNO (3 mg/kg). Mixed effects model, *n =* 6 or 7 animals per group. (**G**) Mean plasma CORT levels in hM4Di+ or mCherry+ animals at stress onset (0 min) and stress termination (30 min) following the intraperitoneal injection of VEH or CNO (3 mg/kg). Two-way ANOVA followed by Fisher’s LSD, *n* = 6 to 9 animals per group. ***P < 0.001

To test the contribution of BLA projection (pyramidal) neurons to stress-induced neuroendocrine activation, we measured plasma CORT following chemogenetic inhibition of BLA projection neurons during stress. We expressed an inhibitory Gi designer receptor exclusively activated by designer drugs (DREADD), hM4Di, or an mCherry control protein within BLA CaMKII (projection) neurons (Fig. 1, A and B), allowing a minimum of 3 weeks for sufficient viral expression and recovery of animals. On the test day, vehicle [VEH; 0.2% dimethyl sulfoxide (DMSO) in saline, 3 ml/kg] or clozapine-*N*-oxide (CNO; 3 mg/kg dissolved at 1 mg/ml) was administered intraperitoneally 30 min before a 30-min restraint episode, and CORT was measured at initiation (*t*_0_) and termination (*t*_30_) of stress (Fig. 1C). To confirm CNO-induced suppression of cells expressing hM4Di in vivo, we collected tissue 90 min following stress onset in a subset of animals to perform immunohistochemistry for visualization of the activity-responsive protein FOS.

CNO reliably reduced the excitability of BLA cells expressing hM4Di but not mCherry cells in the slice of naïve animals (Fig. 1, D and E, and fig. S1, A and B) and reliably reduced FOS expression in response to restraint stress in hM4Di+ cells in vivo (fig. S1, C to G). We also confirmed no effect of CNO on stress-induced CORT release in the absence of hM4Di expression (Fig. 1F and fig. S1H). We therefore conducted subsequent experiments only in hM4Di+ animals using VEH as our control condition and CNO as our experimental condition. Animals administered VEH or CNO displayed no difference in CORT at stress initiation (*t*_0_), and although all animals displayed elevated CORT at stress termination, this was significantly reduced in animals administered CNO (Fig. 1G). These data demonstrate that broad inhibition of BLA pyramidal neurons attenuates stress-induced CORT release.

### Stimulation of BLA projection neurons activates the HPA axis in the absence of stress

**Fig. 2.**
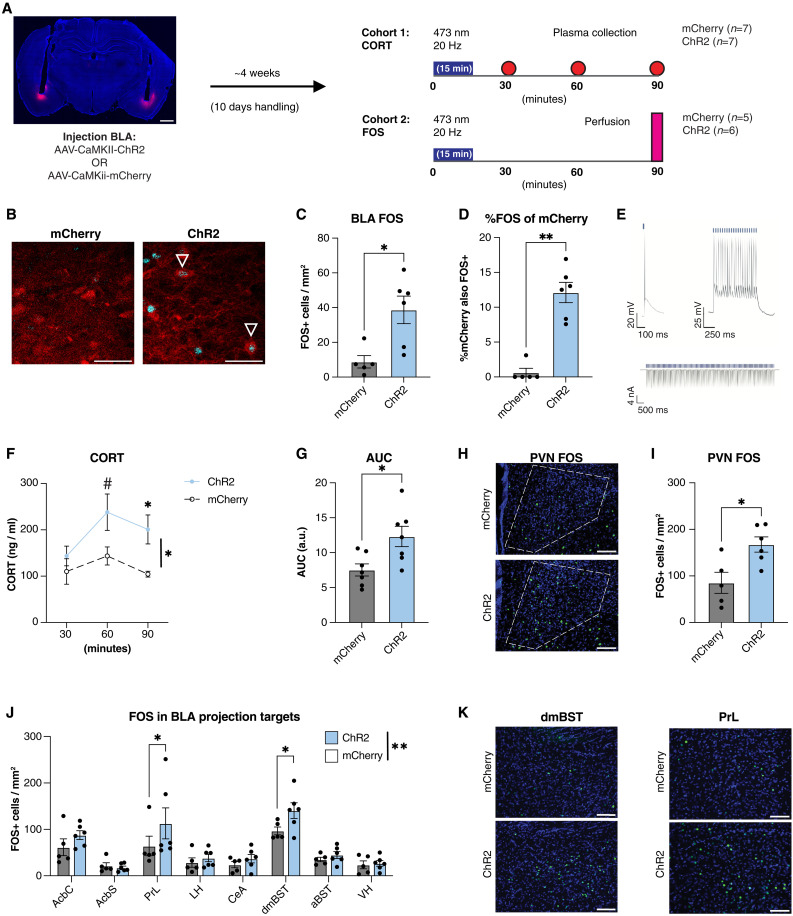
Excitation of BLA projection neurons drives neuroendocrine activation in the absence of stress. (**A**) Representative image of ChR2-mCherry expression in the BLA and overview of experimental procedures. Scale bar, 250 μm. (**B**) Representative images of FOS expression (cyan) in ChR2- or mCherry-expressing cells (red). Scale bars, 50 μm. (**C**) Mean FOS density in the BLA 90 min following photostimulation with 473-nm light (15 min, 20 Hz) in animals expressing ChR2 or mCherry control in the BLA. *t* test, *P < 0.05 *n* = 5 or 6 animals per group. (**D**) Mean percentage of ChR2+ or mCherry+ cells also expressing FOS. Mann-Whitney test, **P < 0.01, *n* = 5 or 6 animals per group. (**E**) Response of a ChR2+ cell in the BLA (top left) to a single pulse of blue light (3 ms) or (top right) to a train of light pulses (20 Hz for 1 s) in current clamp mode; (bottom) response to a train of light pulses (20 Hz for 10 s) in a voltage clamp. (**F**) Plasma CORT in animals following photostimulation of BLA projection neurons versus mCherry control. Two-way ANOVA followed by Fisher’s LSD, *P < 0.05, #*P* = 0.0597, *n* = 7 animals per group. (**G**) AUC between *t*_30_ and *t*_90_ following photostimulation of BLA projection neurons versus mCherry control. *t* test, *P < 0.05; *n* = 7 animals per group. a.u., arbitrary units. (**H**) Representative image of PVN FOS expression 90 min following photostimulation of BLA projection neurons in animals expressing mCherry control (top) or ChR2 (bottom). Scale bars, 100 μm. (**I**) Mean FOS density in the PVN; *t* test, *P < 0.05, *n* = 7 animals per group. (**J**) Mean FOS density in eight BLA target regions following BLA photostimulation. Two-way ANOVA (*P* < 0.01) followed by Fisher’s LSD, *P < 0.05, *n* = 7 animals per group. (**K**) Representative images of FOS expression in the dmBST (left) or PrL (right) in animals expressing mCherry control (top) or ChR2 (bottom). Scale bars, 100 μm.

We next tested whether photostimulation of BLA projection neurons was sufficient to increase plasma CORT in the absence of stress. We virally expressed the light-activated ion channel ChR2 (*72*) or an mCherry control protein in BLA:CaMKII neurons. The laser was reliably positioned to focus photostimulation on the mBA (Fig. 2A and fig. S2A), a BLA subregion we have previously identified as highly sensitive to stress (*19*, *50*). FOS immunohistochemistry and patch-clamp electrophysiology confirmed that photostimulation with 473-nm light increased the activity of BLA neurons expressing ChR2 (Fig. 2, B to E), as previously established (*72*).

To test the effect of BLA photostimulation on HPA axis activation, 473-nm light was delivered through fiber-optic cannulas over 15 min (10 mW; 20 Hz) and plasma collected serially from the lateral tail vein every 30 min (Fig. 2A; cohort 1). Animals expressing ChR2 exhibited greater plasma CORT than those expressing mCherry, particularly 60 and 90 min following the onset of photostimulation (Fig. 2, F and G). As an additional proxy measure of HPA axis activation, we tested in a separate cohort of animals whether photostimulation of BLA pyramidal neurons was associated with the increased activity of PVN neurons. BLA photostimulation increased FOS expression in the PVN (Fig. 2, H and I). Together, this provides strong evidence that activation of the medial BLA is sufficient to drive HPA axis activation even in the absence of a frank stressor.

Given that the BLA does not send any direct projections to the PVN (*3*, *73*), we next wanted to identify candidate brain regions that may relay HPA-activating signals from the BLA to the PVN. We therefore quantified FOS expression in seven downstream brain regions following photostimulation of the BLA: the prelimbic cortex (PrL), nucleus accumbens core (AcbC) and nucleus accumbens shell (AcbS), dorsomedial subdivision of the BST (dmBST) and anterodorsal subdivision of the BST (aBST) [as defined in (*74*, *75*)], the central amygdala (CeA), and the lateral hypothalamus (LH) (fig. S2B). Apart from the nucleus accumbens (NAc), each of these regions relays to the PVN, albeit often via further secondary intermediary relays (*12*, *76*–*80*). Notably, photostimulation of BLA projection neurons increased FOS in multiple downstream brain regions innervated by the BLA (Fig. 2J), specifically the PrL and dmBST (Fig. 2K). Together, these data demonstrate that activation of projection neurons in the BLA drives CORT release in the absence of stress via indirect activation of the PVN, possibly through intermediary regions such as the PrL or dmBST.

### BLA projection neurons innervate diverse downstream regions

**Fig. 3.**
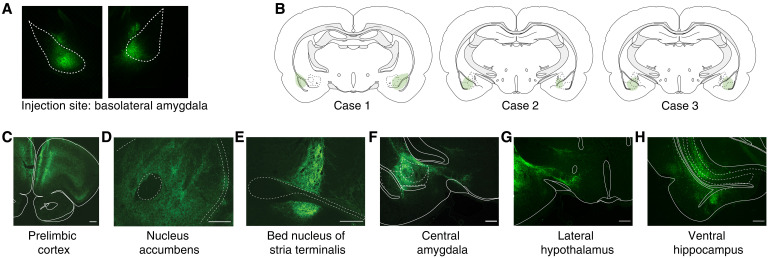
BLA projection neurons innervate diverse downstream regions. (**A**) Representative image of GFP expression following the injection of AAV8-CaMKII-eGFP into the BLA. (**B**) Representative images of the location of injection site for each of the three anatomical cases. (**C** to **H**) Representative images of GFP expression in downstream brain regions following the injection of AAV8-CaMKII-eGFP into the BLA. Images were obtained from different cases and brightness and contrast adjusted independently for each image.

Extensive work has demonstrated that the BLA projects to a wide range of brain regions involved in motivated and aversive behaviors (*15*, *37*, *81*). To replicate these anatomical findings and to guide subsequent experiments, we labeled BLA projection neurons and their fibers by injecting an anterograde virus expressing enhanced green fluorescent protein (eGFP) under the CaMKII promoter into the BLA of three adult male rats (Fig. 3, A and B) and, 3 to 4 weeks later, imaged the expression of eGFP throughout the brain. eGFP was expressed widely throughout many brain regions (fig. S3), replicating many others’ previous anatomical work demonstrating BLA innervation targets (*49*, *73*, *81*–*84*). Notably, we identified strong expression in six limbic regions: the PrL, NAc, BST, CeA, LH, and VH (Fig. 3C), all of which are strongly implicated in motivated behavior or HPA axis regulation [for example, (*8*–*10*, *12*, *85*–*87*)].

### BLA projection neurons are topographically distributed

Projection neuron populations are heterogeneously distributed throughout the BLA (*28*, *48*, *49*). Likewise, we have previously demonstrated that the BLA is not uniformly activated by stress but rather displays gradients of increased stress-induced FOS expression, especially in the medial basal subdivision (*19*). We thus hypothesized that projection populations targeting stress-responsive brain regions would be activated by acute restraint stress and be localized in the mBA. To test this, we examined stress-induced FOS expression in multiple BLA projection populations. We injected cholera toxin subunit B (CTB) bilaterally into one of six target regions: PrL, NAc, BST, CeA, LH, and VH (fig. S4A). One week later, animals were exposed to 30-min restraint stress (stress condition) or remained in their home cage (naïve condition), and brains were then collected 90 min following stress onset and processed for imaging of CTB and FOS. Thus, CTB was used to label a defined projection population, FOS was used to label a cell active during stress exposure, and the presence of both markers within a cell was used to label a cell projecting to the target region and active during stress exposure.

**Fig. 4.**
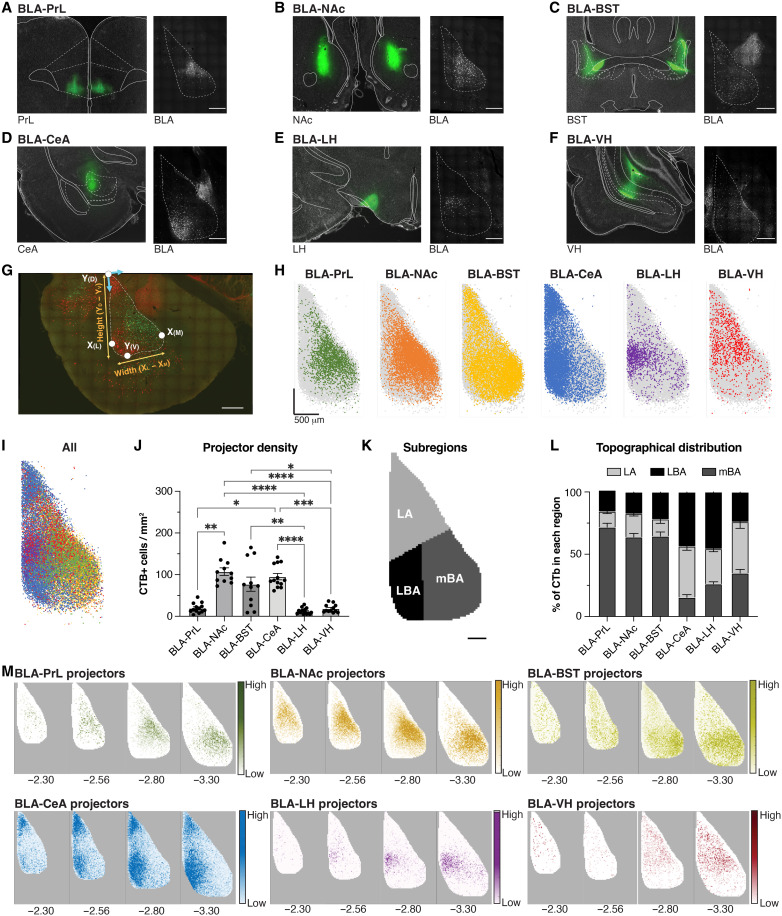
Topographical distribution of BLA projection neurons. (**A** to **F**) Representative images of the injection site of the retrograde tracer CTB (left, green) into the PrL, NAc, BST, CeA, LH, and VH and retrograde expression of CTB in the BLA (right, white). Scale bar, 500 μm. (**G**) Representative image demonstrating normalization. Red, BLA-CeA projectors; green, BLA-NAc projectors; Y_(D)_, most dorsal point; Y_(V)_, most ventral point; X_(M)_, most medial point; X_(L)_, most lateral point. Blue arrows indicate the reference frame. Scale bar, 500 μm. (**H**) Dot plots of normalized coordinates for each CTB+ neuron in the BLA (AP −2.80) for each projection population at AP −2.80. Graphs represent all CTB+ neurons across all animals (green, PrL; orange, NAc; yellow, BST; blue, BST; purple, LH; red, VH; gray, all CTB+ neurons regardless of projection). (**I**) Overlay of all dot plots representing the topographical distribution of BLA neurons projecting to the PrL (green), NAc (orange), BST (yellow), CeA (blue), LH (purple), and VH (red). (**J**) Mean density of CTB+ cells in the BLA following the injection of CTB into the PrL, NAc, BST, CeA, LH, or VH. Kruskal-Wallis test followed by Dunn’s multiple comparisons test, *P < 0.05, **P < 0.01, ***P < 0.001, ****P < 0.0001, *n* = 11 to 15 animals per group. Both CTB-488 and CTB-555 were used to label each projection population and counterbalanced across group. (**K**) BLA subdivision at AP −2.80: LA, LBA, and mBA. Scale bar, 150 μm. (**L**) Mean distribution (%) of CTB-labeled cells in each BLA subdivision for each projection population. Two-way ANOVA followed by Tukey’s post hoc test, *n* = 11 to 15 animals per group. (**M**) Normalized density heatmaps (25-μm by 25-μm pixels) for each projection population across the rostral-caudal axis of the BLA. A darker color represents greater average CTB+ density for each projection population.

We first mapped the location of projection populations within the rostral and intermediate BLA [anteroposterior (AP) −2.12 to −3.30]. We bilaterally injected CTB into each target region and observed the expression of each projection population in the BLA (Fig. 4, A to F). To compare topographical gradients of different projection populations across multiple animals, we localized the coordinates of every CTB+ cell and then normalized these coordinates to the most dorsal point of the BLA and according to a standardized template for each AP position (Fig. 4G). These standardized templates were previously established by our lab using measurements of the BLA taken from 685 slices from 52 age-matched, male rats (*19*) and similar to work by others in mice (*28*, *48*). This visually revealed clear topographical gradients that were distinct between each projection population (Fig. 4, H and I). In addition, we observed significantly greater density throughout the entire BLA (AP −2.12 to −3.60) of BLA-NAc, BLA-BST, and BLA-CeA projection populations than those targeting the PrL, LH, and VH (Fig. 4J). To quantify density gradients throughout the BLA, we subdivided the BLA into the lateral amygdala (LA), lateral basal amygdala (LBA) division, and mBA subdivisions (Fig. 4K), consistent with our recent characterization of stress-induced FOS throughout BLA subregions (*19*). There was a significant location bias for each projection population (Fig. 4L); specifically, the mBA housed most PrL, BST, and NAc projectors and many VH projectors but very few CeA and LH projectors. In contrast, the LBA housed most LH projectors and many CeA projectors, with proportionately few PrL, BST, NAc, or VH projectors. In addition, the LA housed many VH and CeA projectors. Subregion bias remained largely consistent across the rostral-caudal axis (Fig. 4M), although nearly all projection populations had a significantly or near-significantly greater density of expression in caudal sections of the BLA, especially LH and VH projectors (fig. S4, B to G).

Collectively, these findings demonstrate that BLA projection populations are heterogeneous in location bias, with populations targeting the PrL, BST, and NAc as largely intermingled and distinct from populations targeting the CeA and LH. Notably, BLA-PrL, BLA-NAc, and BLA-BST projectors had a significant location bias toward the mBA, the region we have previously identified as being particularly stress-responsive (*19*). This anatomical localization is also consistent with our optogenetic stimulation of the mBA in Fig. 2, which revealed the BST and PrL as two regions most robustly activated by stimulation of these neurons. Thus, their anatomical location suggests that these populations may be more likely recruited by acute psychological stress.

### BLA projection populations are broadly activated by stress in the mBA

**Fig. 5.**
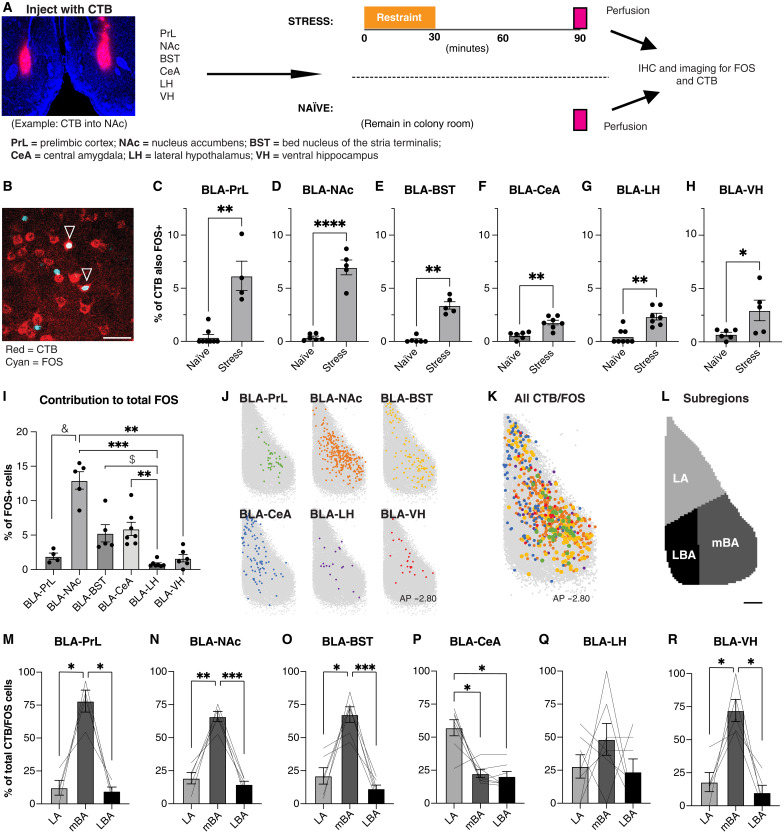
BLA projection populations are broadly activated by stress in the mBA. (**A**) Overview of experimental procedures. IHC, immunohistochemistry. (**B**) Representative image of colocalized expression of FOS (cyan) and CTB (red) in the BLA. Scale bar, 25 μm. (**C** to **H**) Percentage of CTB-labelled cells for each projection population also expressing FOS under the naïve or stress condition. Mann-Whitney or unpaired *t* test, *P < 0.05, **P < 0.01, ****P < 0.0001, *n* = 4 to 8 animals per group. (**I**) Percentage of FOS-labeled cells also expressing CTB for each BLA projection population under the stress condition only. Kruskal-Wallis test followed by Dunn’s multiple comparisons test, $*P* = 0.0813, &*P* = 0.0637, **P < 0.01, ***P < 0.001; *n* = 4 to 7 animals per group. (**J** and **K**) Normalized coordinates of all colabeled CTB+/FOS+ cells for each projection population in the BLA at AP: −2.80 under the stress condition only. (**L**) BLA subregion at AP −2.80: LA, LBA, and mBA. Scale bar, 150 μm. (**M** to **R**) Percentage of total CTB/FOS–colabeled cells for each projection population in each BLA subregion. One-way RM ANOVA followed by Tukey’s multiple comparisons test, *P < 0.05, **P < 0.01, ***P < 0.001, *n* = 4 to 7 animals per group.

To specifically determine whether discrete BLA projection populations were activated by restraint stress, we quantified the percentage of CTB+ cells for each projection population that also expressed FOS (Fig. 5, A and B). Restraint stress (30 min) significantly increased the activation of all projection populations—namely, BLA-PrL, BLA-NAc, BLA-BST, BLA-CeA, BLA-LH, and BLA-VH projectors—as demonstrated by a greater proportion of each projection population also expressing FOS relative to the naïve condition (Fig. 5, C to H). This suggests that although relatively inactive under baseline conditions, a diverse range of BLA projection populations is activated by exposure to acute restraint stress.

To account for differences between BLA projection targets in the total number of cells, we broke down the total number of FOS+ cells depending on their projection target (Fig. 5I). We found that BLA-NAc projectors represented significantly or near-significantly more FOS+ neurons than populations targeting the LH, VH, and PrL, and BLA-CeA and BLA-BST projectors represented significantly or near-significantly more FOS+ neurons than populations targeting the LH. Together, this suggests that despite stress activating multiple projection populations, the largest total number of projection neurons activated by stress targets the NAc, BST, and CeA.

Given previous findings indicating a topographical bias of activation toward the mBA subdivision of the BLA (*19*), we next investigated the anatomical location of CTB+/FOS+ cells to test whether there was also a medial bias despite overall topographical differences for each population. We plotted the normalized coordinates of every colabeled CTB+/FOS+ cell for each projection population (Fig. 5J). This visually revealed a bias in activation toward the LA or mBA among all populations (Fig. 5K). To quantitatively confirm this bias, we divided the BLA into LA, mBA, and LBA subdivisions (Fig. 5L) and quantified the proportion of labeled CTB+/FOS+ cells in each region for each projection population. Most BLA-PrL, BLA-NAc, BLA-BST, and BLA-VH projection populations activated by stress were located within the mBA (Fig. 5, M to O and R), generally consistent with their topographical distribution. Notably, there was a topographical bias toward the LA in activated BLA-CeA projectors (Fig. 5P), and there was no topographical bias in activated BLA-LH projectors (Fig. 5Q) despite both populations exhibiting a high percentage of projectors in the LBA. Together, this confirms that despite differences in the topographical organization of discrete projection populations, acute restraint stress activates a diverse array of projection populations that are commonly located with a bias toward the mBA. This is especially prominent for diffuse populations such as the CeA, where only a subpopulation of BLA-CeA projectors located in the dorsal LA appeared to be activated by stress.

### Inhibition of discrete BLA projection populations does not influence the stress-induced CORT response

**Fig. 6.**
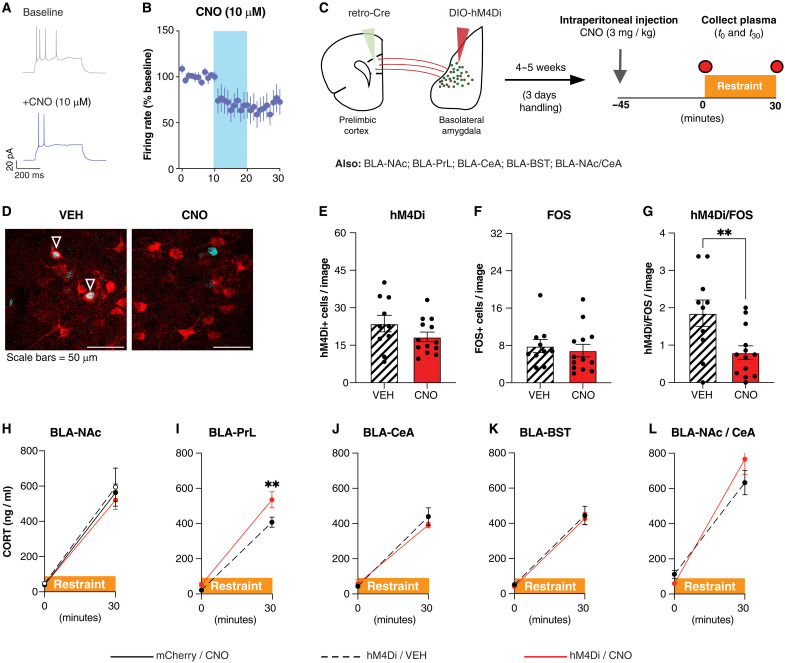
Individual effect of BLA projection populations on stress-induced CORT release. (**A**) Representative traces of evoked action potentials before (baseline) and during the application of CNO (10 μM). (**B**) Time plot demonstrating that the bath application of CNO (10 μM, blue band) reduces firing rates in BLA neurons expressing hM4Di. (**C**) Overview of experimental procedures. (**D**) Representative image of hM4Di-mCherry (red) and FOS expression (cyan) in the BLA of animals expressing hM4Di-mCherry in BLA-NAc projection neurons and injected intraperitoneally with VEH (left) or CNO (3 mg/kg; right) 45 min before stress exposure. Tissue was collected 90 min following stress onset. Triangles indicate colocalization. Scale bars, 50 μm. (**E**) Mean number of hM4Di+ cells per slice. *t* test, *n* = 10 to 13 animals per group. (**F**) Mean number of FOS+ cells per slice. Mann-Whitney test, *n* = 10 to 13 animals per group. (**G**) Mean number of colocalized hM4Di/FOS+ cells per slice. Mann-Whitney test, **P < 0.01 *n* = 10 to 13 animals per group. (**H**) Plasma CORT levels immediately at stress onset (0 min) and stress termination (30 min) in animals expressing hM4Di or mCherry in BLA neurons projecting to the NAc, following the injection of VEH or CNO (3 mg/kg) 45 min before stress onset. Mixed effects analysis, *n* = 10 to 14 animals per group. (**I** to **L**) Plasma CORT levels immediately at stress onset (0 min) and stress termination (30 min) in animals expressing hM4Di- or mCherry in BLA neurons projecting to the PrL (I), CeA (J), BST (K), or both the NAc and CeA (L), following the injection of VEH or CNO (3 mg/kg) 45 min before stress onset. Mixed effects analysis or two-way ANOVA followed by Sidak’s post hoc comparisons test, **P < 0.01, *n* = 6 to 10 animals per group.

Excitotoxic lesions (*55*, *56*), pharmacological modulation of discrete neurochemical systems (*57*–*60*), or nonspecific chemogenetic inhibition of BLA projection neurons (Fig. 1F) attenuates stress-induced CORT. It is unknown, however, whether these effects are driven by the collective action of multiple projection populations or whether specific BLA projection populations independently contribute to stress-induced HPA axis activation, as has been reported for many stress-related behavioral end points such as anxiety-like behavior or social avoidance (*38*, *41*). We therefore used an intersectional viral approach (*88*) to express the inhibitory Gi DREADD hM4Di in discrete BLA projection populations by injecting a retrograde virus expressing Cre into a target region [PrL, NAc, BST, or CeA: AAV2(rg)-eSYN-EGFP-T2A-iCre-WPRE)] and a Cre-dependent virus expressing hM4Di or mCherry into the BLA (AAV8-hSyn-DIO-mCherry or AAV8-hSyn-DIO-hM4Di-mCherry; fig. S5A). We then allowed 4 to 5 weeks for sufficient viral expression and recovery of the animals. AAV8-hSyn-DIO-hM4Di-mCherry into the BLA without the injection of the retrograde Cre virus into a target region did not yield any expression of hM4Di-mCherry (fig. S5B), confirming the cre dependence of the virus and therefore allowing the restriction of hM4Di expression to a discrete projection population. Using the BLA-NAc projection population as a model, patch-clamp electrophysiology and FOS immunohistochemistry confirmed that administration of CNO inhibits the activity of BLA neurons expressing hM4Di (Fig. 6, A to G, and fig. S5, C to E), as previously established (*88*).

We then tested whether inhibition of independent BLA projection neuron populations reduces stress-induced CORT, as global BLA:CaMKII inhibition did. On the experiment day, animals were injected with either CNO (3 mg/kg, intraperitoneally) or VEH (3 ml/kg in 0.2% DMSO) 45 min before 30-min restraint stress, and plasma was collected for measurement of CORT at initiation (*t* = 0; baseline) and termination of stress (*t* = 30; peak) (Fig. 6C). In animals expressing hM4Di in BLA-NAc projectors, there were no significant differences in basal or stress-induced CORT between animals expressing the functional DREADD and administered either CNO or VEH and in animals expressing mCherry controls and administered CNO (Fig. 6H). No difference between either control groups (mCherry/CNO versus hM4Di/VEH) again confirmed that CNO has no effect on plasma CORT in the absence of a functional DREADD, and we therefore performed all subsequent experiments with only two groups: hM4Di/VEH and hM4Di/CNO. In addition, CNO administration in animals expressing hM4Di in BLA-NAc projectors had no effect on basal or stress-induced CORT compared to either control group, thus also demonstrating that inhibition of BLA-NAc projectors had no effect on stress-induced CORT.

Using the same approach, we then tested the impact of chemogenetic inhibition of three other BLA projection populations: BLA-PrL, BLA-CeA, and BLA-BST. Complementing our prior topographical findings using CTB, each projection population visually displayed differences in density and topography of viral expression within the BLA (fig. S5B). Unexpectedly, inhibition of BLA-PrL projectors potentiated stress-induced CORT (Fig. 6I), suggesting that this circuit may endogenously function to restrain or inhibit HPA activation during stress. In contrast, there was no effect on stress-induced CORT when hM4Di was expressed in either the BLA-CeA or BLA-BST projection populations (Fig. 6, J and K). Last, we hypothesized that chemogenetic inhibition of two projection populations in tandem may recapitulate the HPA-dampening effects of large-scale inhibition of the BLA. To this extent, we chose two projection populations that we had demonstrated were robustly activated by stress and were expressed widely throughout the BLA. Thus, we restricted hM4Di to both BLA-NAc and BLA-CeA projections in the same animal by injecting a retrograde virus expressing Cre into both the NAc and CeA and a Cre-dependent virus expressing hM4Di into the BLA. Again, we observed no difference between CNO- or VEH-treated animals in stress-induced CORT (Fig. 6L).

Collectively, these data demonstrate that chemogenetic inhibition of individual—or even dual—BLA projection neuron populations alone does not recapitulate the HPA-dampening effect of chemogenetic inhibition of projection neurons within the BLA together. Furthermore, inhibition of BLA-PrL projectors enhanced stress-induced CORT, suggesting that this projection may have an endogenous stress-dampening role.

## DISCUSSION

### Summary of findings

Despite a clear role in processing fear and anxiety-like behavior (*37*, *89*), the functional organization of diverse BLA circuits involved in stress-induced activation of the neuroendocrine response is unclear. Here, we established four major findings in adult male rats: (i) BLA projection neurons are both necessary and sufficient for stress-induced HPA axis activation; (ii) projection neuron populations are topographically distributed across expression gradients within the BLA; (iii) diverse projection populations targeting the PrL, NAc, BST, CeA, LH, and VH are activated by exposure to acute psychological stress, with a primary location bias in the medial BLA; and (iv) inhibition of singular BLA projections does not recapitulate effects of global inhibition of BLA projection neurons. Together, this suggests that a network of BLA projection populations, primarily located in the mBA, is broadly activated by acute psychological stress and collectively contributes to the regulation of the HPA axis.

### Necessity and sufficiency of mBA projection neurons in HPA axis activation

The BLA is robustly activated by psychological stressors (*16*, *17*) and widely implicated in stress-related responses such as avoidance behavior and memory (*37*, *89*). Evidence for the contribution of the BLA to the endocrine stress response, however, is inconsistent (*55*–*71*), possibly due to the heterogeneity of BLA neurons in responding to stimuli of both positive and negative valences (*27*, *37*). Previous work has pointed toward the mBA as especially sensitive to stress (*19*). We therefore targeted this region for chemogenetic and optogenetic manipulation specifically; thus, while others demonstrate mixed effects on BLA contribution to HPA axis activation through less specific approaches such as electrical stimulation, lesions, or γ-aminobutyric acid type A agonists (*55*, *56*, *64*–*71*), our effects may have been apparent only by excluding potentially stress-inhibiting cell types such as astrocytes or inhibitory interneurons (*90*, *91*) by limiting expression to the CaMKII promoter and by targeting our manipulations to a defined stress-sensitive subregion such as the mBA.

### Topographical patterns of stress-induced FOS expression

The notion that the medial aspects of the BLA are particularly stress-sensitive (*19*) is supported by our topographical mapping of BLA projection neurons activated by stress. Although there are diverse expression gradients of each projection population across the BLA, most populations displayed a bias in stress-induced activation toward the mBA or LA, even in populations that displayed high density within the LBA such as BLA-CeA and BLA-LH projectors. Collectively, this positions the mBA and LA as especially involved during acute stress.

Given that many BLA-CeA projectors in lateral regions were not activated by stress, it remains unclear what distinguishes anatomically distinct BLA-CeA subpopulations to support a bias in stress-induced activation, as well as what sort of stimuli leads to activation of the CeA projectors located in the LBA. Given the critical role of the BLA-CeA circuit in the expression of learned fear (*92*), BLA-CeA projectors in the LBA may be preferentially activated with repeated (rather than acute) presentation of aversive stimuli to drive a learned response. Beyeler *et al.* (*28*) have shown that BLA-CeA projectors are activated during exposure to learned, aversive stimuli such as quinine but not to learned, rewarding stimuli. Thus, BLA-CeA projectors may become active with repeated exposure to an aversive stimulus (i.e., as learning occurs), and greater activation may reflect enhanced learning. Alternatively, molecularly distinct subpopulations of BLA-CeA projectors have been identified by Kim *et al.* (*45*) to differentially drive approach or avoidance behaviors. As such, it is apparent that distinct subpopulations exist within larger projection populations, which may be further distinguished on the basis of anatomical location or molecular identity.

### Topographical distribution of BLA projection neurons

Topographical mapping of projection neurons revealed distinct expression gradients within the BLA for each population. BLA populations targeting the PrL, NAc, and BST were predominantly located in the mBA, which we previously identified as particularly responsive to stressors (*19*). In contrast, CeA projectors were more commonly found in the LA and LBA and had significantly less expression in the mBA. Last, VH projectors were predominantly located in the LA. This heterogeneity strongly agrees with previous work mapping the topography of projection neurons in rodents (*47*, *48*), most notably work by Beyeler *et al.* (*28*) and Reppucci and Petrovich (*49*), and reiterates the anatomical diversity of the BLA. This heterogeneity likely contributes to the capacity of the BLA to influence a diverse array of behavioral (*37*) and physiological outcomes (*25*, *55*). There are likely also heterogeneity and specificity of inputs to the BLA; for example, previous work has identified cholinergic (*93*) and noradrenergic inputs (*94*) that preferentially target the basal amygdala, while corticotropin-releasing hormone receptor 1 is preferentially expressed in the LA (*50*, *95*, *96*). It will be important to continue investigating whether these inputs synapse onto discrete projection populations or cell types, particularly ones that are also responsive to stressors, or whether they broadly target many different cells in that region.

### Network of BLA projection neuron populations activated by stress

Our colocalization experiments revealed stress-induced activation of multiple BLA projection neuron populations. Activation of BLA-VH, BLA-PrL, BLA-BST, and BLA-CeA projectors was expected, as many of these populations and targets are known to drive stress-related processes, including avoidance behavior and memory consolidation (*37*, *97*–*100*). In contrast, activation of BLA-NAc projectors by stress was unexpected given that this circuit is readily self-stimulated in rodents (*42*, *101*) and has been shown to counteract stress-induced anxiety (*43*). However, this circuit has also been shown to mediate the glucocorticoid enhancement of memory (*102*), suggesting a potential role in salience encoding. As such, it would be informative to test whether activation of this circuit increases with progressively more salient stimuli, such as increasingly stronger foot shocks. In addition, distinct subsets of BLA-NAc projectors expressing unique molecular markers have been demonstrated to facilitate anxiogenic changes following chronic social defeat stress (*46*, *103*, *104*), and thus, a subset of this projection population may be involved in aversive behavioral changes. Thus, it is essential to consider that within individual projection populations, there may be further heterogeneity dependent on precise anatomical targeting or molecular identity.

### Network of BLA projection neuron populations involved in HPA axis regulation

While broad inhibition of CaMKII neurons in the BLA targeting multiple projection populations inhibited stress-induced CORT, inhibition of singular projection neuron populations in the BLA targeting the CeA, BST, NAc, or a combination of CeA and NAc had no effect on stress-induced CORT release. There are several possible explanations. First, our electrophysiological recordings demonstrated that CNO-mediated inhibition of hM4Di+ neurons was more robust when the expression occurred broadly in CaMKII BLA populations (Fig. 1E and fig. S1, A and B) than when it was cre-dependently restricted to the BLA-NAc projection population (Fig. 6B and fig. S5, C and D). This suggests possible technical limitations of using projection-specific constructs, namely incomplete inhibition of hM4Di+ neurons, which was not as apparent when hM4Di was broadly expressed in CaMKII BLA neurons. However, we confirmed CNO-mediated inhibition of projection-specific hM4Di+ BLA populations both in vivo (Fig. 6G) and in slice (Fig. 6B), establishing the functionality of the tool. Given that CNO also reliably suppresses the synaptic transmission of hM4Di+ cells independent of, and in addition to, inhibition of neuronal electrical activity (*105*), we expect the impact of these tools to effectively inhibit circuit transmission beyond only reducing neuronal activity. Other labs have observed strong functional effects using these same projection-specific constructs despite an incomplete inhibition of neural firing (*88*). We ourselves observed a functional impact of BLA-PrL inhibition (Fig. 6I). Nonetheless, we cannot fully rule out the notion that a lack of observed effect on inhibiting discrete populations may be a result of incomplete inhibition, however, and that circuit-specific effects may instead only be observed using tools that lead to a more complete inhibition of neural activity.

A second possible reason we did not observe an effect of inhibiting discrete BLA populations is that we only selected a subset of circuits for testing. We targeted circuits projecting to the BST, PrL, NAc, and CeA on the basis of dense innervation by the BLA and—apart from BLA-NAc, which we selected as a negative control—a known role in HPA axis regulation (*79*). Yet, the BLA projects to other intermediary regions that relay to the PVN, including the medial amygdala, lateral septum, and substantia innominata (*76*, *106*–*108*). These circuits were not directly investigated. Although these projections are relatively sparse, we also cannot rule out the notion that inhibition of these singular BLA projections may have a significant impact on stress-induced HPA axis activation. This remains to be investigated.

Last, given the evolutionary importance of reliably mounting a neuroendocrine response to stress, multiple redundant circuits are likely responsible for stress-induced HPA axis activation. In general, diverse cortical, mnemonic, and sensory inputs converge at multiple relay points such as the dorsomedial hypothalamus, nucleus of the solitary tract, PrL, VH, and BST to regulate HPA axis activation (*4*, *12*, *76*). Each of these regions receives input from the BLA, either directly or indirectly via an intermediary relay. With this perspective and given its rich connectivity with diverse regions at multiple relay points, the BLA likely has an important “coordinative,” modulatory role in HPA axis regulation. As such, at least in terms of HPA axis activation, disruption of a singular BLA projection may have a limited impact on HPA axis activation because of alternative (i.e., redundant) activating inputs to the PVN from multiple independent, extra-BLA structures. Instead, the impact of the BLA may be unmasked only with broad disruption of diverse BLA projection populations collectively targeting multiple circuits converging at multiple downstream relay points. This would together allow the activity of multiple structures to be simultaneously dampened, ultimately leading to an additive effect at each relay point that combined may lead to a functional, measurable impact. Given the lack of effect we observed following singular inhibition of three major BLA projection populations (BLA-NAc, BLA-CeA, and BLA-BST) but a robust effect following inhibition of BLA CaMKII neurons generally, we expect this to be the most likely explanation.

Notably, this redundancy may not similarly apply to HPA axis inhibition. The BLA is uniquely anatomically well situated for a bidirectional influence on the HPA axis but via distinct pathways. For instance, the BLA has access to pathways that ultimately activate the PVN via the CeA, medial amygdala, and BST, each of which has divergent and independent relays to the PVN, such as the nucleus tractus solitarius, BST, medial preoptic area, dorsomedial hypothalamus, or peri-PVN (*12*, *76*, *77*). In contrast, the BLA also has access to “negative feedback” pathways to the PVN, which inhibit HPA axis activity, via projections to the PrL and VH, but which both use a specific subregion of the BST as a common inhibitory relay to the PVN (*8*, *9*). As such, limbic inhibition of the PVN may more readily involve the activation of a common inhibitory relay in the BST, while limbic activation of the PVN may involve more diffuse, parallel, and complementary relays to the PVN. The only functional impact of chemogenetic inhibition of singular BLA projections we observed was via the BLA-PrL circuit, which enhanced HPA axis activation. This suggests that singular activation of the BLA-PrL endogenously acts to constrain HPA axis activity, consistent with findings implicating the PrL in HPA axis inhibition (*9*, *109*, *110*). This may have especially important implications in stress adaptation such as during reexposure to stressful contexts (*111*).

As such, our overall concluding perspective is that the BLA bidirectionally influences HPA axis regulation, with HPA axis stimulation mediated by broad activation targeting diffuse, parallel circuits and HPA axis inhibition mediated by more singular activation targeting defined, common inhibitory relays. Notably, this may be why broad pharmacological approaches such as those targeting the endocannabinoid system may be particularly effective in mediating the HPA response to stress (*58*, *59*), as the endocannabinoid system is widely expressed throughout the entire BLA (*112*) and may be capable of influencing (i.e., “dampening”) multiple projection populations simultaneously. Similarly, specific molecular markers may be common to multiple projection populations; for example, *Thy1+* neurons in the BLA strongly project to the BST, PrL, and NAc while largely sparing projections to the CeA (*33*). It will thus be essential to continue investigating common, tractable molecular markers of stress-activated projection neuron populations and whether they act in combination to influence BLA-driven HPA axis activity.

## MATERIALS AND METHODS

### Animals

All animal protocols were approved by the University of Calgary Animal Care Committee (AC24-0088 and AC20-0090) and followed guidelines from the Canadian Council on Animal Care. Adult male Sprague Dawley rats were obtained from Charles River Laboratories (175 to 225 g upon arrival; strain code 001) and maintained under a 12-hour light-dark cycle (lights on at 8 a.m.) with food and water available ad libitum. All experiments were performed between 8 a.m. and 2 p.m. within the light phase of the cycle. Animals were pair housed and randomly assigned to a given treatment group, although cage mates were always in identical treatment groups and underwent all aspects of experimentation at the same time, including intraperitoneal injections, blood collection, stimulus exposure, and tissue collection. For optogenetic experiments, animals were single housed for a minimum of 2 weeks before testing. Experiments were run as single cohorts or waves of cohorts of experimental animals, and aside from specific control experiments (ensuring that CNO in the absence of the DREADD receptor did not influence the HPA axis), no experiments were run in duplication once data were unblinded and analyzed.

### Stereotaxic surgery

Rats were maintained under isoflurane anesthesia and analgesic treatment [meloxicam (2 mg/kg, subcutaneously)] in the stereotaxic apparatus (Stoelting). Coordinates targeting brain regions are described in the Supplementary Materials (table S1), and all injections of virus, anatomical tracer, or cannulation were bilateral. A viral vector or CTB was delivered in a glass capillary carefully lowered into the brain and pressure injected using a NanoInject II apparatus (Drummond Scientific). CTB was delivered in 13.8 nl of boluses every 30 s, and viruses were delivered in 27.6 to 69.0 nl of boluses every 20 to 45 s. After the final target volume was reached, the capillary remained in place for ~10 min following the delivery of the final bolus to allow for the diffusion of the agent. For optogenetic experiments, 2 weeks were allowed for recovery before bilateral implantation of a 600-μm-diameter mono fiber-optic cannula [Doric Lenses; numerical aperture (NA), 0.48] 0.1 mm above the injection site and secured with METABOND, dental cement, and four anchoring screws. To allow for the sufficient recovery of the animal and the expression of the virus or tracer, behavioral testing began a minimum of 7 days following the injection of CTB or securing of the ferrule and a minimum of 3 weeks following the injection of virus.

### Cholera toxin subunit B (CTB)

Work was guided by McGarry and Carter (*48*). Dilutions (0.2%) of Alexa-conjugated CTB in 0.04 M phosphate-buffered saline (PBS; CTB-488 or CTB-555; Invitrogen) were injected bilaterally into one of six target regions: PrL (303.6 nl), VH (303.6 nl), NAc (303.6 nl), LH (303.6 nl), BST (193.2 nl), and CeA (151.8 to 193.2 nl). Every animal received injections in two different brain regions using different colors in each region (e.g., CTB-555 delivered bilaterally to CeA and CTB-488 delivered bilaterally to NAc), but each projection population was analyzed independently and only after verification of CTB in the target region. To eliminate imaging bias for CTB, the tracer color was counterbalanced such that a similar number of animals received an injection of CTB-555 or CTB-488 for each projection population. A minimum of 7 days was allowed for recovery from surgery before experiments began.

### Viral vectors

Constructs were diluted in 0.01 M sterile PBS to reach the desired titer. For CaMKII chemogenetic inhibition, AAV8.CaMKII.hM4Di.mCherry [1.6 × 10^13^ to 2.5 × 10^13^ genome copies (GCs)/ml] was injected into the BLA (Addgene viral prep no. 50477-AAV8; http://n2t.net/addgene:50477; RRID:Addgene_50477; gift from B. Roth). For CaMKII optogenetic excitation, AAV5-CaMKII-hChR2(H134R)-mCherry (4.6 × 10^12^ to 6.7 × 10^12^ GCs/ml) was injected into the BLA (Addgene viral prep no. 26975-AAV5; http://n2t.net/addgene:26975; RRID:Addgene 26975; gift from K. Deisseroth). For CaMKII viral controls, AAV8.CaMKII.mCherry (1.3 × 10^13^ to 6.7 × 10^13^ GCs/ml) was injected into the BLA (Neurophotonics, Universite Laval). For projection-specific chemogenetic inhibition, AAV8-hSyn-DIO-hM4D(Gi)-mCherry (1.9 × 10^13^ GCs/ml) was injected into the BLA (Addgene no. 44362; gift from B. Roth), and AAV2(retro)-eSYN-EGFP-T2A-iCre-WPRE (0.9 × 10^13^ to 1.2 × 10^13^ GCs/ml) was injected into either the PrL, NAc, BST, or CeA (Vector Biolabs). For projection-specific viral controls, AAV8-hSyn-DIO-mCherry (1.6 × 10^13^ GCs/ml) was injected into the BLA (Addgene no. 50459; gift from B. Roth), and AAV2(retro)-eSYN-EGFP-T2A-iCre-WPRE (0.9 × 10^13^ to 1.2 × 10^13^ GCs/ml) was injected into the NAc (Vector Biolabs). For anterograde CaMKII tracing experiments, AAV5.CaMKII-eGFP was injected into the BLA (Universite Laval). Stereotaxic coordinates are listed in table S1.

### Chemogenetic inhibition

Chemogenetics were used to inhibit CaMKII-expressing or projection-specific (BLA-PrL, BLA-NAc, BLA-BST, BLA-CeA, and BLA-NAc/CeA) neurons in the BLA. Following a recovery period to allow the expression of hM4Di (or mCherry control) in CaMKII-expressing or projection-specific neurons in the BLA, animals were habituated to a test room for 1 hour/day for 3 days before the experiment day and handled by the experimenter for 2 min in the position used for injections. On the experiment day, animals were injected intraperitoneally in their colony room with either CNO (Cayman Chemical; 3 mg/kg at 3 ml/kg), to inhibit hM4Di+ neurons, or VEH (0.2% DMSO in 0.9% saline) and, 30 min later, were moved to a separate testing room and immediately placed into clear plastic restraint tubes for 30 min. For projection-specific chemogenetic experiments, animals were injected 45 min before stress, as this time point has been demonstrated to exhibit the strongest inhibition for these constructs (*88*). The experimenter was blinded to the viral condition of the animal [mCherry versus hM4D(Gi)]. Blood samples were collected immediately at initiation (*t* = 0) and termination (*t* = 30) of restraint stress. Following stress exposure, animals returned to their home cage and remained in the testing room. Ninety minutes following stress onset, animals were anesthetized and perfused, and brains were collected and processed for imaging.

### Optogenetic stimulation

Optogenetics were used for photostimulation of CaMKII neurons in the BLA. Following a recovery period to allow the expression of ChR2 (or mCherry control) in CaMKII neurons in the BLA, animals underwent extensive handling and habituation to the experimenter and optic ferrules. On days 1 to 6, animals were carted to the testing room each morning, handled for 1 min each, and then placed into an empty cage with only bedding for ~90 min. On days 7 to 10, the same procedure occurred, but animals were also habituated to the optic fiber (no light) attached to their heads while exploring their test cage. Habituation and testing cages had a custom-made lid with a slit cut in it to allow for the entry of the fiber.

On the experimental day, animals were carted to the testing room 1 hour before testing. A light source (473 nm, Laserglow Technologies LRS-0473-GFM-00100-05 LabSpec 473 nm DPSS Laser System connected to Laser Power Supply PSU-III-LED) was connected to the implanted ferrules with a fiber-optic cable (600-μm core diameter, Doric Lenses) attached to a beam splitter. The lasers were controlled using the open-source programmable pulse generator Pulse Pal (*113*). Blue light (5-ms-long pulses;10-mW laser intensity measured at the tip of optic ferrule) was shone at 20 Hz for 15 min. Light intensity was measured before experimentation using a Standard Photodiode Power Sensor (ThorLabs S120C) connected to a Compact Power and Energy Meter Console (ThorLabs PM100D). The experimenter was blinded to the viral condition (mCherry versus ChR2) of the animal. The animal remained connected to the fiber for an additional 15 min (30 min in total), then removed, and returned to their home cage. For the investigation of the effects of BLA photostimulation on the CORT response (experiment 1: mCherry, *n* = 7; ChR2, *n* = 7), blood samples from the lateral tail vein were collected to measure plasma CORT 30, 60, and 90 min following the onset of 473-nm stimulation. Animals were anesthetized and perfused, and brains were collected for the verification of viral expression. For the investigation of the effects of BLA photostimulation on FOS expression (experiment 2: mCherry, *n* = 5; ChR2, *n* = 6), a new set of animals was used, and no blood samples were collected in this cohort. Rather, 90 min following the onset of 473-nm stimulation, animals were anesthetized and perfused, and brains were collected to verify the viral expression and perform immunohistochemistry for FOS.

### Restraint stress for projection-specific FOS labeling

To label discrete projection neurons, CTB-488 or CTB-555 was injected into target regions of the BLA (PrL, NAc, BST, CeA, LH, or VH) in a counterbalanced manner. Animals were allowed to recover for a minimum of 7 days before testing. On the experiment day, animals were carted from their colony room to the adjacent experimental room and placed into a clear Plexiglas restraint tube for 30 min. Animals were then returned to their home cage with their cage mate and remained in the testing room until 90 min following stress onset, where they were immediately anesthetized and transported to a nearby room for perfusion and brains were collected. Control animals remained in the colony room immediately until the time of euthanasia. Brains were then processed for imaging.

### Blood collection and CORT analysis

Animals were gently placed into clear Plexiglas restraint tubes, and blood samples were collected into ice-chilled, EDTA-treated microcuvettes (Sarstedt AG & Co. KG; no. 16.444.100) from a small nick over the lateral tail vein. Tail blood was centrifuged at 10,000 rpm for 20 min at 4°C to separate plasma, which was stored at −20°C until CORT analyses. Plasma samples were analyzed with an enzyme-linked immunosorbent assay (ELISA) kit (Arbor Assays; no. K014-H5) by following the manufacturer’s instructions and as performed previously (*114*). Standards were run in triplicate, and samples were tested in duplicate and diluted 1:100 to ensure that levels fit the standard curve. Groups were compared at individual time points or as an area under the curve (AUC). The following formula for the AUC was used: {[(*t*_30_ + *t*_60_) * 30 min]/2} + {[(*t*_60_ + *t*_90_) * 30 min]/2} (*115*). Animals were excluded if the sufficient volume of plasma was not collected to run the ELISA. One animal was excluded because of pipetting error during the ELISA. Grubb’s test was performed to identify any blood samples that were an outlier within their group, and these animals were excluded for analyses at all time points.

### Tissue collection

Brains were collected and processed identically at the termination of all experiments. Animals were anesthetized with an overdose of sodium pentobarbital and transcardially perfused with 0.9% saline (~60 ml per rat, 30 ml/min) followed by 3.8% paraformaldehyde in 0.01 M PBS (~120 ml per rat, 30 ml/min). Following perfusion, brains were removed, immersed in 3.8% paraformaldehyde in 0.01 M PBS overnight before being switched to a 20% sucrose solution in PBS for 48 to 72 hours, and then transferred to a 30% sucrose solution in PBS for cryoprotection. Coronal sections (40 μm) were cut in a one-in-four series on a Leica SM 2010R sliding microtome and stored at −20°C in antifreeze until further analyses [30% (w/v) sucrose, 1% (w/v) polyvinylpyrrolidone-40, 30% (v/v) ethylene glycol, and 0.0065% (w/v) sodium azide in PBS; adapted from (*116*)].

### Electrophysiology

Whole cell patch-clamp electrophysiology used to validate functional hM4Di and ChR2 expression is described in Supplementary Methods.

### Histology

To verify CTB or viral expression at the completion of each experiment, free-floating sections were rinsed for 3 × 10 min in PBS, mounted onto charged slides, and coverslipped using Fluoroshield with 4′,6-diamidino-2-phenylindole (DAPI) mounting medium (Sigma-Aldrich). Images of hM4Di, ChR2, or mCherry expression were acquired with a Leica DM4000 B light-emitting diode (LED) microscope using a 2.5×/0.07-NA PLAN (Leica 556036), 5×/0.12-NA PLAN EPI (Leica 566076) objective, or 10×/0.30-NA HCX PL FLUOTAR (Leica 506507) or an Olympus VS120 slide scanner using a 10×/0.4-NA air objective. Imaging parameters (i.e., exposure and intensity) remained identical for all images being directly compared, except for mCherry controls; this fluorophore was observed to have significantly greater fluorescence intensity than hM4D(Gi)-mCherry, and we therefore used lower intensity and exposure settings. The location of maximal expression of virus and the location of cannula tips were plotted onto coronal images adapted from an atlas (*117*). Animals were excluded if there was no expression in at least one hemisphere, significant expression outside of the BLA, significant damage at the injection site, or placement of the cannula tip outside of the BLA. Images were prepared by ImageJ for observation and analysis.

### Anterograde tracing

Brains were collected 3 to 4 weeks following the surgical delivery of AAV-CaMKII-eGFP into the BLA. Only animals with the majority of cell body expression within the BLA were included for analyses. Forty-micrometer-thick sections were observed in a one-in-four series throughout the entire brain (approximately AP +4.00 to −8.00), and eGFP expression was manually recorded by the experimenter. Select images representing notable regions with green fluorescent protein (GFP) expression were acquired at 2.5×, 5×, and 10× magnifications on a Leica DM 4000B LED. Images were prepared by ImageJ, and brightness and contrast were adjusted for each image independently.

### FOS immunohistochemistry

Immunohistochemistry was performed identical to previous studies (*19*, *118*). Free-floating sections of the BLA were first rinsed for 3 × 10 min in PBS followed by 3 × 10 min in 0.1% PBST (PBS and Triton X-100, 0.1%). Sections were then blocked for 1 hour at room temperature with gentle agitation in 5% normal donkey serum in PBS and incubated for 23 hours at 4°C in an antibody diluent with an anti-cFos antibody raised in rabbit (cFos, Cell Signaling Technology no. 2250s; 1:300 for colocalization with CTB; 1:400 for all other experiments). Following a 3 × 10 min wash in PBST, sections were incubated for 2 hours at room temperature with a donkey anti-rabbit Alexa Fluor 647 (Alexa-647)– or Alexa Fluor 488 (Alexa-488)–conjugated secondary antibody (Alexa-647, Jackson ImmunoResearch no. 711-605-152; Alexa-488, Jackson ImmunoResearch no. 711-545-152; 1:100 for colocalization with CTB; 1:125 for all other experiments). The antibody diluent was composed of 0.1% (v/v) Triton X-100, 0.1% (w/v) bovine serum albumin, 0.05% (w/v) sodium azide, and 0.04% (w/v) sodium EDTA in PBS. Last, sections were rinsed for 3 × 10 min in PBST and 2 × 10 min in PBS, mounted onto charged slides, and coverslipped using Fluoroshield with DAPI mounting medium (Sigma-Aldrich).

### FOS colocalization with ChR2, hM4Di, or mCherry in the BLA

For in vivo validation of optogenetic and chemogenetic experiments, we quantified the coexpression of FOS with viral protein in the BLA following experimental procedures. After performing immunohistochemistry for FOS, images of the BLA were acquired using a Leica TCS SPE II confocal microscope using a 20×/0.55-NA HC PL FLUOTAR objective. Parameters (i.e., exposure and intensity) remained identical for all images being directly compared, except for mCherry controls; this fluorophore was observed to have significantly greater fluorescence intensity than hM4D(Gi)-mCherry, and we therefore used lower intensity and exposure settings. Only animals with bilateral expression of viral protein or ferrule within the BLA were included for analysis, and images were collected from both hemispheres in regions with maximal viral expression. Images were only collected if there was the presence of viral protein, thus resulting in variance between animals in the number of images collected (validation of CaMKII-hM4Di: *n* = 8 to 14 slices per animal from three or four animals per group; validation of CaMKII-ChR2: *n* = 3 to 9 slices per animal from five or six animals per group; validation of DIO-hM4Di: *n* = 3 to 8 slices per animal from 10 to 13 animals per group). FOS, ChR2-mCherry, hM4Di-mCherry or mCherry fluorophore, and DAPI signals were acquired independently and exported to ImageJ for quantification. The number of cells expressing FOS was counted automatically by ImageJ using identical detection parameters for all images being directly compared, and hM4Di-mCherry, ChR2-mCherry, or mCherry or colocalization of either signal with FOS was determined manually. Colocalization was determined as a FOS signal bound by the expression of viral protein. The experimenter was blinded to the condition of each animal throughout quantification.

### FOS quantification following optogenetic stimulation of the BLA

After immunohistochemistry for FOS, a one-in-four series of 40-μm-thick sections was collected, and images were acquired for each animal on a Leica DM4000 B LED microscope using a 20×/0.55-NA HC PL FLUOTAR objective. DAF and L5 filter cubes were used to detect the expression of DAPI (to label cell bodies) and Alexa-488 (to label FOS), respectively. Images were acquired in defined regions of interest across the rostral-caudal axis of each brain region (Fig. 2H and fig. S2B). Only animals with bilateral placement of optic ferrules within the BLA were included for analysis (*n* = 5 or 6 animals per group), and only slices with an intact region of interest were acquired. Quantified regions included the PVN (*n* = 2 to 6 images per animal), PrL (*n* = 12 to 14 images per animal), AcbC (*n* = 8 to 10 images per animal), AcbS (*n* = 8 to 10 images per animal), dmBST (*n* = 6 to 8 images per animal), aBST (*n* = 8 to 10 images per animal), CeA (*n* = 5 to 12 images per animal), LH (*n* = 15 to 20 images per animal), and VH (*n* = 5 to 8 images per animal). All images from regions being directly compared were imaged at the same time using identical imaging parameters (i.e., exposure and intensity). The number of cells expressing FOS was counted automatically using ImageJ. The experimenter was blinded to the condition of each animal throughout quantification. One animal was excluded from all analyses as Grubb’s test identified outlier FOS values in three of nine regions quantified.

### Topographical mapping and quantification of CTB+ and FOS+ cells

#### Overview

The experimenter remained blinded to the conditions of each animal during imaging, plotting, and counting. Although all animals received counterbalanced CTB-488 injections into one target region and CTB-555 injections into a second target region, analyses of projection populations within the same animal were performed independently. Only images with tissue undamaged throughout processing, sectioning, and immunohistochemistry were included. In total, 5 to 16 slices (mean: 11 slices) per animal were analyzed from 11 to 15 animals per projection population (with 4 to 7 animals per condition). Following imaging, we normalized all FOS+ and CTB+ neurons to a standardized shape of the BLA to more accurately observe density gradients across the BLA. Analyses were largely guided by work from Beyeler *et al.* (*28*) and using standardized BLA templates established by our prior work (*19*). These prior templates were established through measurements of the BLA across the rostral-caudal axis, taken from 685 slices from 52 age-matched, male rats.

#### Imaging

Immunohistochemistry for FOS expression was performed, and 20× tile scan images of the BLA from AP −2.30 to −3.30 [Paxinos and Watson atlas (*119*)] were acquired using a Leica DM4000 B LED microscope equipped with a 20×/0.55-NA HC PL FLUOTAR objective and appropriate lasers and filters to visualize DAPI, CTB-488, CTB-555, and Alexa-647 (for FOS). Each projection population was imaged independently with different parameters (i.e., exposure and light intensity) but remained constant across all images of the same projection population.

#### Detection and plotting of CTB and FOS labels

We used Imaris software to semiautomatically detect CTB+ and FOS+ cells of a standardized size and quality using the spot detection function. Detection parameters remained identical between images labeling the same projection. However, because of differences in average FOS expression between each experimental run, we adjusted detection parameters for FOS detection between experiments in attempt to keep basal FOS numbers consistent. All CTB- and FOS-labeled cells were localized to the most dorsal point of the BLA (Fig. 4G), and coordinates were then normalized to standardized BLA templates established previously (*19*) and according to the rostral-caudal position assigned to each image. These templates included the division of the BLA into the LA, mBA, and LBA, as defined previously (*19*) and approximating the divisions by Paxinos and Watson (*119*). Normalization procedures are described in further detail within Supplementary Methods.

#### Quantification

All quantification was performed following the normalization of coordinates to standardized BLA dimensions. Density quantification was calculated as the total number of cells counted in region/total area of BLA or subregion. Colocalization experiments were calculated for each animal as (i) the percentage of total CTB cells labeling a specific projection also expressing FOS and (ii) the percentage of total FOS cells also expressing a CTB cell labeling a specific projection. Rostral-caudal differences were quantified by comparing the mean CTB density of sections containing slices from AP −2.30 and −2.56 (rostral) to those from AP −2.80 and −3.30 (caudal). One animal was excluded in all FOS analyses because of the complete absence of FOS expression but was included for CTB topography analyses. One animal was excluded in all FOS/CTB colocalization analyses because of the expression of fewer than 30 CTB+ cells in total. Two animals were excluded when calculating the percentage of colocalized cells in each subregion as a result of having the complete absence of colocalized cells. Two animals were excluded when calculating rostral-caudal differences because of the absence of intact caudal slices.

#### Representation

In Figs. 4H and 5J, normalized coordinates for all labeled cells from all animals were plotted as gray onto the same graph, and only those labeling a single projection were pseudocolored. In Figs. 4I and 5K, normalized coordinates for all labeled cells were plotted onto the same graph, and each individual projection was pseudocolored a different color. In Fig. 4M, heatmaps representing the average density of CTB+ within 25-μm by 25-μm bins were generated using a custom MATLAB script.

### Statistics

GraphPad Prism 10 software was used for all statistical analyses. The group size was selected on the basis of our previous studies (*19*, *50*, *114*). The number of rats per group is represented by *n*, and *N*/*n* is the number of neurons/rat in electrophysiology experiments. The declared group size is the number of independent values, and statistical analysis was done using these independent values. All statistical significance was set at *P* < 0.05. A Shapiro-Wilk test was performed to assess normality for all data; if data failed these assumptions, nonparametric tests were conducted. When comparing the means of two independent groups, an unpaired *t* test was performed (for nonparametric: Mann-Whitney test). When comparing the means of more than two independent groups, a Kruskal-Wallis test was conducted followed by Dunn’s multiple comparisons test. When comparing the means from two dependent groups, a paired *t* test was used (for nonparametric: Wilcoxon matched-pairs signed-rank test). When comparing the means of related measures within a single group, a one-way repeated measures (RM) analysis of variance (ANOVA) was performed followed by Tukey’s multiple comparisons test. When comparing the means of two or more groups on a related measure, a two-way RM ANOVA or mixed-effects model was carried out followed by Tukey’s multiple comparisons test (when comparing data within a condition) or Fisher’s least significant difference (LSD; when comparing data between conditions). For further statistical details regarding each analysis, see tables S2 to S19. Error bars in figures represent the means +/− SEM.
