# Supplementary material for: A network of basolateral amygdala projection neurons contributes to stress-induced activation of the hypothalamic-pituitary-adrenal axis
Source: Sci Adv. 2025 Nov 7;11(45):eadv3737. doi: 10.1126/sciadv.adv3737 (PMC12588290; doi:10.1126/sciadv.adv3737)
Supplement: Supplementary file 1 — Figs. S1 to S5 Supplementary Methods Tables S1 to S19 [file sciadv.adv3737_sm.pdf]

Science Advances
AAAS

Supplementary Materials for

A network of basolateral amygdala projection neurons contributes to stress-induced activation of the hypothalamic-pituitary-adrenal axis

Robert J. Aukema *et al.*

Corresponding author: Stephanie L. Borgland, mnhill@ucalgary.ca

Sci. Adv. **11**, eadv3737 (2025)
DOI: 10.1126/sciadv.adv3737

This PDF file includes:

Figs. S1 to S5
Supplementary Methods
Tables S1 to S19

Supplementary Figure 1

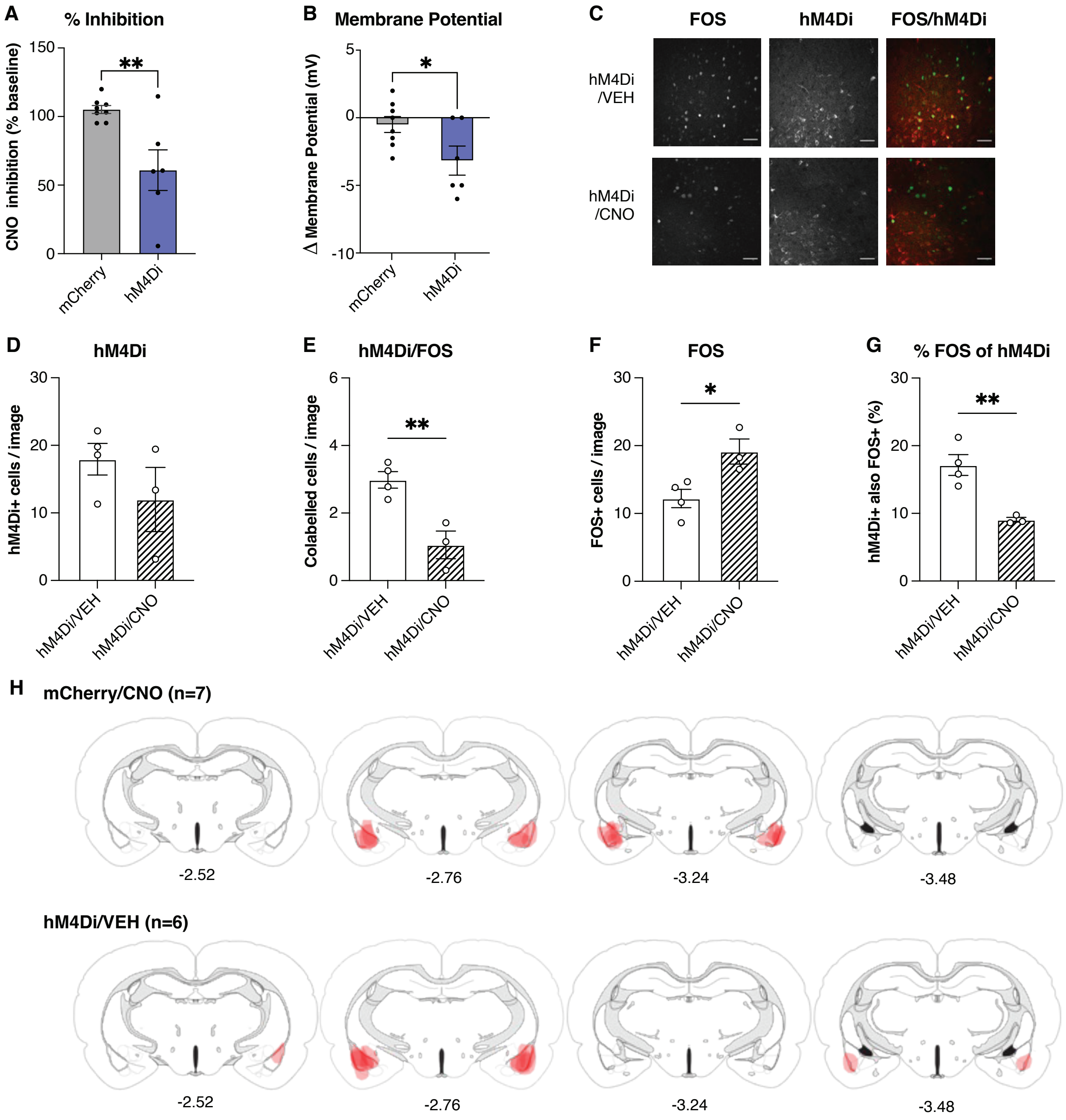

Supplementary Figure 1. Chemogenetic inhibition of the BLA.
(A) CNO (10 μM) significantly reduced firing rates in BLA cells expressing hM4Di compared to mCherry controls. *t* test, $^{**}p<0.01$; *N/n* =8/3 (mCherry) and 6/4 (hM4Di) cells/animal. **(B)** CNO (10 μM) significantly increased membrane hyperpolarization compared to baseline. *t* test, $^{*}p<0.05$; *N/n* =8/3 (mCherry) and 6/4 (hM4Di) cells/animal. **(C)** Representative image of FOS (green), hM4Di (red), and co-expression following administration of VEH or CNO (3 mg / kg; *i.p.*) 30 min prior to restraint stress; tissue collected 90 min following stress onset; scalebar, 50 μm. **(D)** Mean number of hM4Di+ cells per slice. *t* test; n=3-4 animals per group. **(E)** Mean number of hM4Di+/FOS+ co-labelled cells per image. *t* test, $^{**}p<0.01$; *n*=3-4 animals per group. **(F)** Mean number of FOS+ cells per slice. *t* test, $^{*}p<0.05$; *n*=3-4 animals per group. **(G)** Mean percentage of hM4Di+ cells also expressing FOS. *t* test, $^{**}p<0.01$; *n*=3-4 animals per group. **(H)** Representative images of hM4Di or mCherry expression in the BLA; red indicates area of maximal expression for each animal, overlaid among all animals.

Supplementary Figure 2

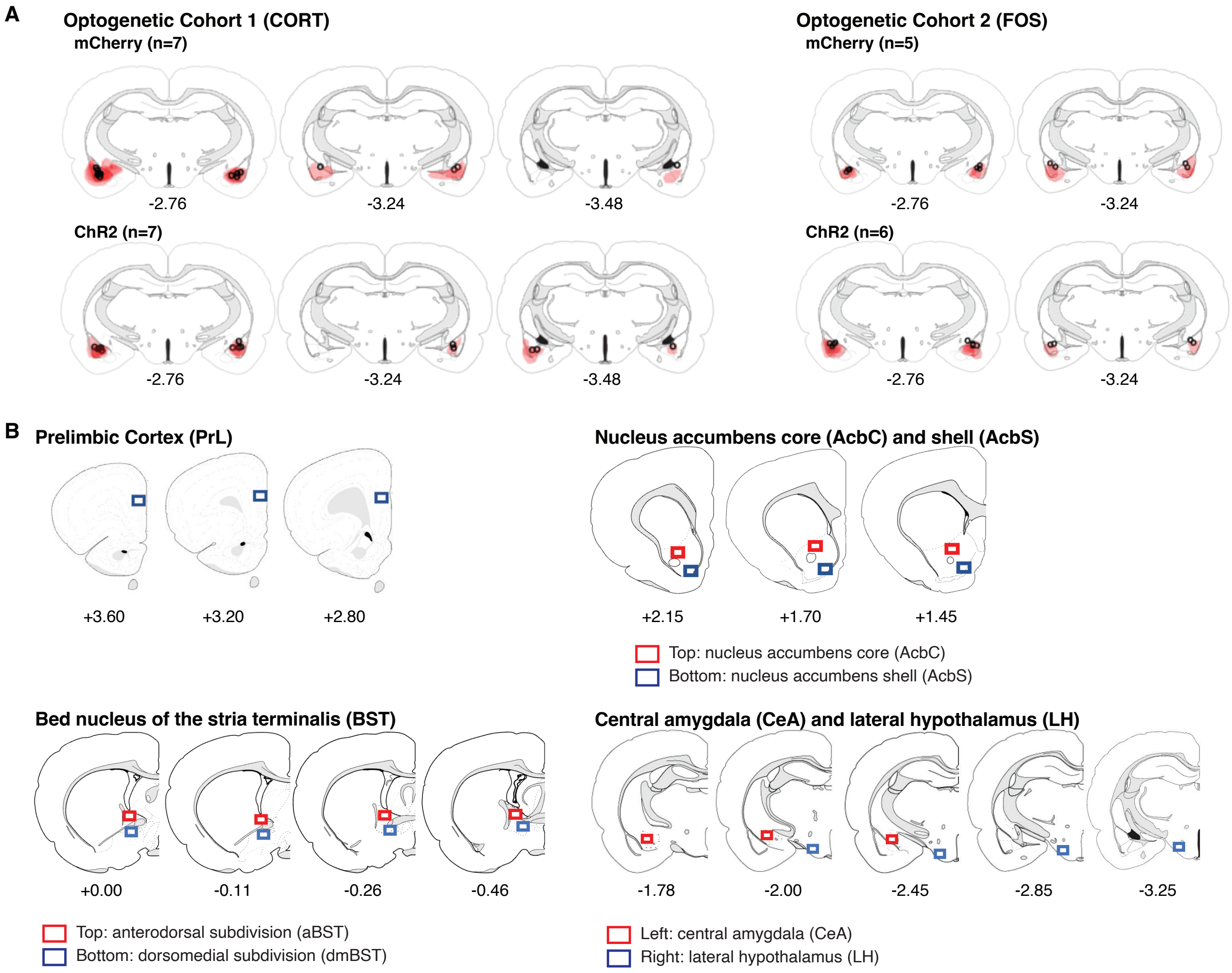

Supplementary Figure 2. Optogenetic stimulation of the BLA.
(A) Representative images of mCherry (*top*) or ChR2 (*bottom*) expression in the BLA; red indicates area of maximal expression for each animal, overlaid among all animals; black circles indicate location of ferrule tip. **(B)** Representative regions-of-interest where cells were counted in the prelimbic cortex (PrL), nucleus accumbens core (AcbC) and shell (AcbS), dorsomedial bed nucleus of the stria terminalis (dmBST), anterodorsal bed nucleus of the stria terminalis (adBST), central amygdala (CeA), and lateral hypothalamus (LH). Distance is from bregma.

Supplementary Figure 3

Presence of eGFP expression in various brain regions following injection of AAV8-CaMKII-eGFP into the BLA

Region	Case 1	Case 2	Case 3
Orbitofrontal cortex	X	X	X
Prelimbic cortex	X	X	X
Insular cortex	X	X	X
Anterior cingulate cortex	X	X	X
Infralimbic cortex	X	X	X
M2	X	X	X
Accumbens shell	X	X	X
Accumbens core	X	X	X
Olfactory tubercle / piriform cortex	X	X	X
Dorsal striatum	X	X	X
Bed nucleus of the stria terminalis	X	X	X
Claustrum	X	X	X
Paraventricular thalamus	X	X	X
Nucleus of the lateral olfactory tract		X	X
Substantia innominata	X	X	X
Central Amygdala		X	X
Medial amygdala		X	X
Lateral hypothalamus	X	X	X
Entorhinal cortex		X	X
Zona incerta		X	
vCA1		X	X

Supplementary Figure 3. Targets of BLA projection neurons.

Supplementary Figure 4

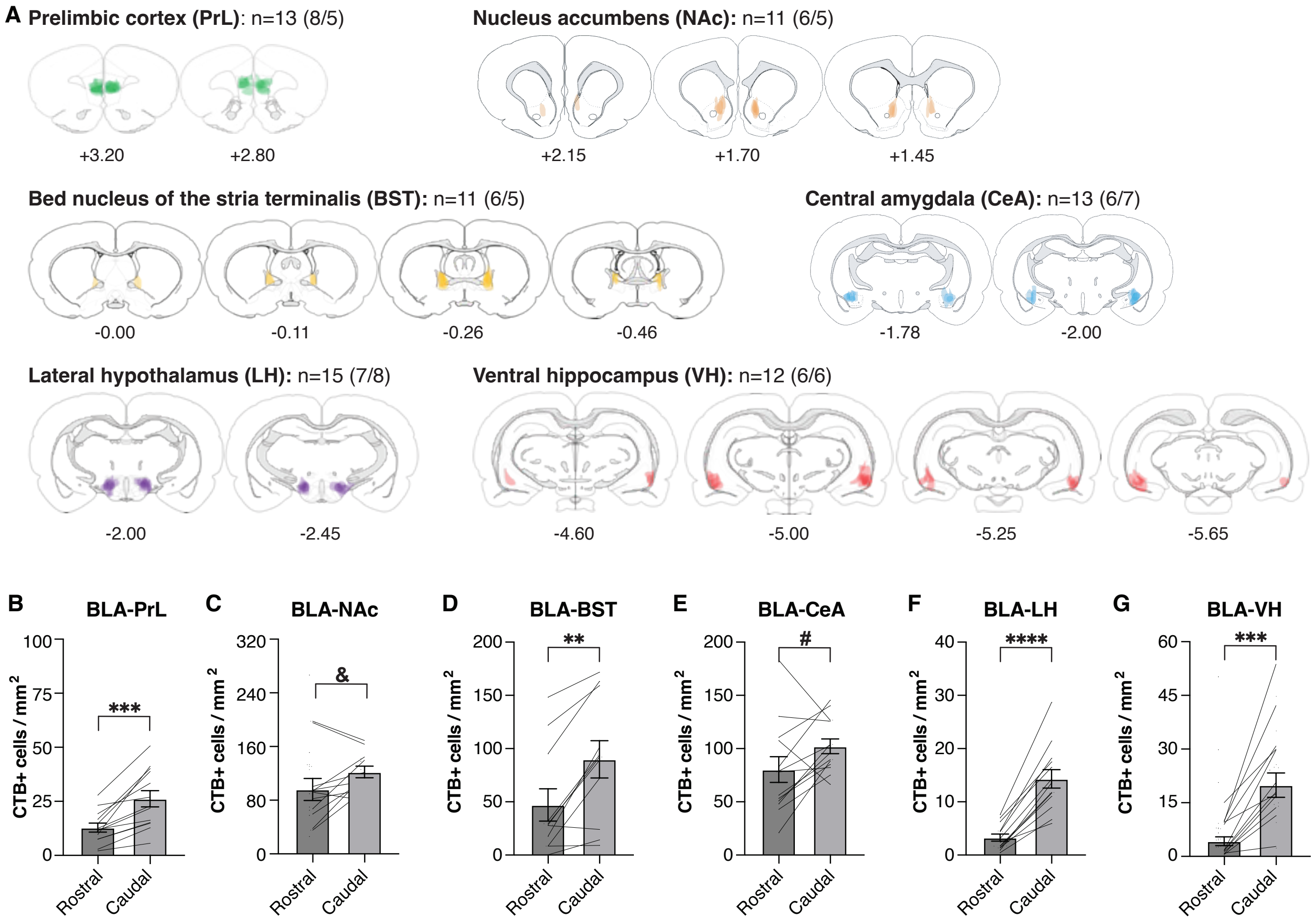

Supplementary Figure 4. Location of CTB injections and rostral-caudal distribution

(A) Representative images showing location of CTB injection in the prelimbic cortex (PrL; n=13 animals (naïve: n=8; stress: n=5)), nucleus accumbens (NAc; n=11 (6/5)), bed nucleus of the stria terminalis (BST; n=11 (naïve = 6/ stress = 5)), central amygdala (CeA; n=13 (6/7)), lateral hypothalamus (LH; n=15 (7/8)), or ventral hippocampus (VH; n=12 (6/6)). Coloured spread indicates area of maximal expression of CTB at the injection site. Distance is from bregma. **(B-G)** Mean density of CTB-labelled cells in the rostral (AP -2.30 and -2.56) vs. caudal (AP -2.80 and -3.30) sections of the BLA. Wilcoxon matched-pairs signed rank test or *t* test, &p=0.0531, #p=0.0577, **$p<0.01$, ****$p<0.0001$; n=11-13 animals per group. All data in Figure 4 was quantified in sections from AP -2.30 to AP -3.30 and after normalization of coordinates to the most dorsal point of the BLA and standardized dimensions and shape of the BLA.

Supplementary Figure 5

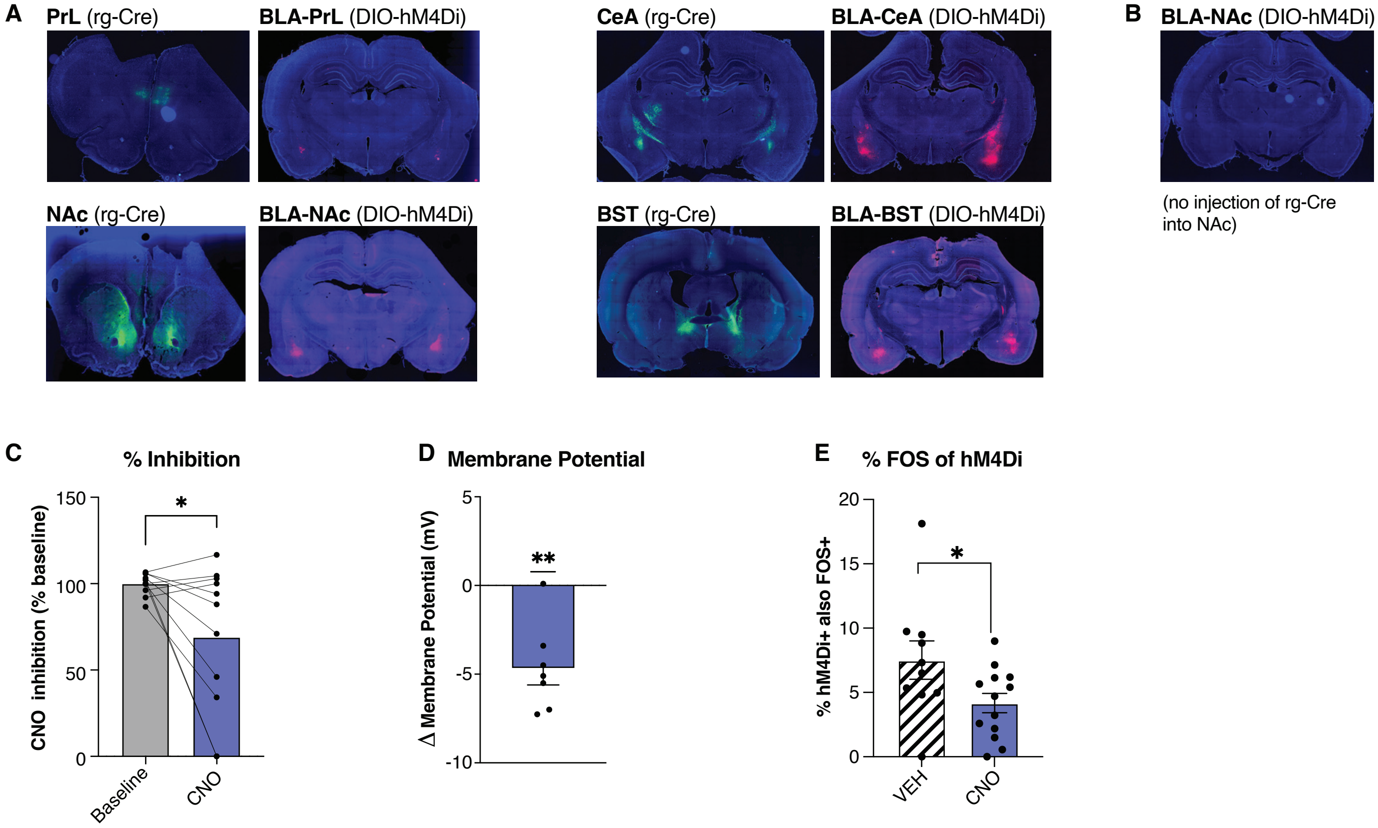

Supplementary Figure 5. Projection specific chemogenetic inhibition of discrete BLA circuits. **(A)** Representative images of rg-Cre-eYFP expression in target region (green; *left*), and DIO-hM4Di-mCherry expression in the BLA (red; *right*). Brightness and contrast adjusted independently for each image **(B)** Representative image of DIO-hM4Di-mCherry expression in the BLA without any injection of rg-Cre-eYFP in a target region. **(C)** CNO (10 μM) significantly reduced firing rates in cells expressing hM4Di compared to baseline. Paired t test, $*p<0.05$; $N=11$ animals. **(D)** Change in membrane potential from baseline to 18min following CNO bath application. One sample t-test, $p=0.0026$. **(E)** Mean percentage of colocalized mCherry/FOS+ cells per animal, following administration of VEH or CNO (3 mg / kg; *i.p.*) 45 min prior to restraint stress; tissue collected 90 min following stress onset. Unpaired t-test, $**p<0.01$; $n=10$-13 animals per group.

SUPPLEMENTARY METHODS

Topographical mapping of CTB+ neurons

Detection and plotting of CTB and FOS labels. We used Imaris software to semi-automatically detect CTB+ and FOS+ cells of a standardized size and quality using the spot detection function. For all images, CTB labeling was detected having an average diameter of 12 μm, and the quality was manually set for each image until most labelled cells were identified by the software, as determined by the experimenter. Mis-labelled cells were manually removed, and non-labelled cells identified by the experimenter were manually added using the spot detection function. For FOS labeling, the spot detection function identified any FOS labeling averaging 7 μm diameter. The quality parameters remained identical for images labeling the same projection. However, due to differences in average FOS expression between each experimental run, we adjusted the quality parameters for FOS detection between experiments in attempt to keep basal FOS numbers consistent; thus, minimum quality ranged from 8.5-11.5 across the entire experiment, with no limits on maximum quality.

Normalization. For each image, we identified its approximate rostral-caudal position according to the Paxinos & Watson atlas (-2.30, -2.56, -2.80, -3.30; (119)) and measured the height (dorsal-ventral axis), width (medial-lateral axis), and triangular area of the BLA **(Fig. 4G)** using Imaris Cell Imaging Software (Oxford Instruments). The formula for triangular area was: [(height x width) / 2]. Any images with a BLA triangular area exceeding two times the standard deviation of the mean calculated from other images within the same projection assigned a similar plane were re-assigned to a plane that was more in line with the calculated triangular area.

Previously, we measured the average width, height, and triangular area of the BLA at 4 defined anterior-posterior (AP) positions (19) and rounded to the nearest 25um for standardization: AP -2.30 (width = 825 μm; height = 1775 μm); AP -2.56 (width = 900 μm; height = 1950); AP -2.80 (width = 1175 μm; height = 2200 μm); AP -3.30 (width = 1375 μm, height = 2425 μm). To subdivide the BLA into the LA, LBA, and mBA subdivisions and to account for the curvature of the BLA along the fiber tracts, we established a standardized shape derived from the Paxinos & Watson atlas (119) that fit manually identified BLA neurons, comprising 25 μm x 25 μm "pixels" used for representation and quantification. Data used to establish these standardized templates are described in detail by Aukema *et al,* 2024 (19).

We then normalized coordinates of FOS+ and CTB+ neurons from each image according to these standardized dimensions. For each raw coordinate, the x-coordinate was normalized to the average width of the BLA at that rostral-caudal position, and the y-coordinate to the average height of the BLA at that rostral-caudal position. This established new (x,y) coordinates that maintained their original relative position in the BLA but could now be directly compared to images with a BLA of different raw dimensions.

Quantification. To visually represent gradients of density across the BLA, the average density of FOS+ neurons were calculated per 25um x 25um bin and represented in heatmaps using a custom MATLAB script. Each pixel value represents average density per image, averaged across animals in the same group. To compare group differences, we calculated FOS density for each animal individually and then compared group means. The total area of a subregion was calculated as: [(number of 25 μm x 25 μm pixels comprising the subregion of interest) * 25 μm * 25 μm]. The density was then calculated as: [total # of FOS+ cells detected in all pixels comprising the subregion of interest / total area of subregion of interest]. As multiple images were often collected from each AP plane for each animal, data were analyzed as average density per image: [(total # of cells from all pixels comprising the subregion of interest, from all slices) / (total area of subregion of interest * number of slices)]. These calculations were streamlined using a custom MATLAB script that can be accessed at the authors' request.

Electrophysiology. For slice experiments, animals were deeply anaesthetised with isoflurane, decapitated, and coronal slices (300 μm) containing the BLA were cut using a vibratome (VT1200, Leica Microsystems) in room temperature, NMDG-based artificial cerebrospinal fluid (ACSF). Slices were then briefly transferred to a submerged chamber containing NMDG ACSF of the following composition: 93 mM NMDG, 2.5 mM KCl, 1.2 mM NaH_2PO_4, 30 mM $NaHCO_3$, 20 mM HEPES, 25 mM glucose, 2 mM thiourea, 5 mM Na-ascorbate, 3 mM Na-pyruvate, 0.5 mM $CaCl_2 \cdot 4H_2O$ and 10 mM $MgSO_4 \cdot 7H_2O$, where they were maintained at 32°C for 10-12 mins to allow for protective recovery of tissue. Finally, slices were transferred to a holding chamber containing regular ACSF of the following composition: 126mM NaCl, 2.5mM KCl, 1.4 mM NaH_2PO_4, 1.2 mM $MgCl_2$, 2.4 mM $CaCl_2$, 11 mM glucose and 25 mM $NaHCO_3$; equilibrated with 95% O_2 and 5% CO_2. When time to record, slices were individually transferred to a chamber on an upright microscope (Olympus BX51) and superfused continually with ACSF (32°C, flow rate: 2.0

ml min-1). Neurons were visualised with a 40X water-immersion objective using infra-red Dodt-tube gradient contrast optics.

Functional DREADD expression and blue light photostimulation in BLA pyramidal neurons was confirmed using *ex vivo* patch clamp electrophysiology. Using fluorescence, neurons expressing CaMKII-hM4Di were identified by the presence of an mCherry reporter. Whole-cell current-clamp recordings (Axopatch 700B, Molecular Devices) were then conducted in labelled neurons using a K-Gluconate-based internal solution containing: 130 mM K-Gluconate, 10 mM HEPES, 10 mM KCl, 4 mM Mg2ATP, 0.3 mM Na2GTP and 10 mM Na2-Phosphocreatine (pH 7.3). Neurons were maintained at a holding membrane potential of -70 mV by current injection through the patch-electrode. Action potential firing was evoked once every 60 seconds by a series of 5 incremental depolarizing current pulses (5-25 pA increments, 250 ms duration). In each neuron, the magnitude of current injection was adjusted such that ~ 3-4 action potentials were elicited during the 2nd-4th steps. To determine DREADD functionality, Clozapine-N-oxide (CNO, 10 μM) was bath applied, and the same amount of current was injected as baseline to assess change in neuronal excitability. To confirm that blue light photostimulation reliably excited BLA ChR2 cells *ex vivo*, single pulses and trains were elicited using a 470 nm Light Emitting Diode (LED) (405 mW: Thorlabs) through a 40X microscope objective. We examined the response(s) to (i) a single pulse of blue light (3 ms) in current and voltage clamp mode, (ii) a 20Hz train for 1 s in current and 10 s in voltage clamp mode, and (iii) a 500 ms light pulse in voltage clamp was reliably repeated in several different brain slices from BLAChR2 expressing rats.

Table S1. Summary of stereotaxic coordinates and injections.

Construct	Region	Volume (nl)	AP (mm)	ML (mm)	DV
AAV-eGFP<br>AAV-ChR2<br>AAV-mCherry<br>AAV-DIO-mCherry<br>AAV-DIO-hM4Di<br>AAV-CaMKII-mCherry<br>AAV-CaMKII-hM4Di	BLA	138-207<br>828<br>828<br>515.2<br>515.2<br>220.8-276<br>220.8-276	-2.8	±4.9 to 5.0	-8.4 to -8.7
CTB-488<br>CTB-555	PrL	303.6	+3.0	±0.5	-4.1
	VH	303.6	-5.3	±5.4	-7.0 to -7.2
	NAc	303.6	+1.7	±1.4	-7.1
	LH	303.6	-2.7	±1.5	-8.8 to -9.3
	BST	193.2	-0.3	±1.7	-7.1
	CeA	151.8-193.2	-2.2	±4.0	-8.1
AAV-Cre (rg)	PrL<br>NAc<br>BST<br>CeA	386.4nl<br>579.6nl<br>386.4nl<br>386.4nl	+3.0<br>+1.7<br>-0.3<br>-2.1	±0.5<br>±1.4<br>±1.7<br>±2.3 (12° outward)	-4.1<br>-8.6<br>-7.1<br>-8.1

Table S2. Summary of statistical tests used

	Test Used		n			Descriptive stats		p-value		DEGREES OF FREEDOM & F/t/z/R/ETC VALUE	
Figure Number	Which test?	Section & paragraph #	Exact value	Defined	Section & paragraph #	Reported	Section & paragraph #	Exact value	Section & paragraph #	Value	Section & Paragraph #
1F	Mixed-effects analysis	Fig. Leg.	6,7	Animals per group<br><br>(1-2 samples/ animal)	Fig. Leg.	Error bars are mean +/- SEM	Results, "Statistics"	p=0.9431	-	F(1,11) =0.0053	-
1G	2Way ANOVA<br><br>Fisher's LSD	Fig. Leg.	6,9	Animals per group	Fig. Leg.	Error bars are mean +/- SEM	Results, "Statistics"	**Condition:** p=0.0043<br>**Time:** p<0.0001<br>**Interaction:** p=0.0102	-	F(1,13)=11.89<br>F(1,13)=58.57<br>F(1,13)=8.997	-
2C	Unpaired t-test<br><br>Passed normality	Fig. Leg.	5,6<br><br>(3-9)	Animals per group<br>(slices per animal)	Fig. Leg<br><br>(Methods)	Error bars are mean +/- SEM	Results, "Statistics"	p=0.0108	-	t(9)=3.203	-
2D	Mann-Whitney test<br><br>Failed normality	Fig. Leg.	5,6<br><br>(3-9)	Animals per group<br>(slices per animal)	Fig. Leg<br><br>(Methods)	Error bars are mean +/- SEM	Results, "Statistics"	p=0.0043	-	-	-
2F	2Way ANOVA<br><br>Fisher's LSD	Fig. Leg.	7, 7	Animals per group	Fig. Leg.	Error bars are mean +/- SEM	Results, "Statistics"	**Condition:** p=0.0104<br>**Time:** p=0.0526<br><br>**Interaction:** p=0.3504	-	F(1,12)=9.208<br>F(1.738, 20.86)=3.554<br>F(2,24)=1.096	-
2G	Unpaired t-test<br><br>Passed normality	Fig. Leg.	7,7	Animals per group	Fig. Leg.	Error bars are mean +/- SEM	Results, "Statistics"	p=0.0154	-	t(12)=2.823	-
2I	Unpaired t-test<br><br>Passed normality	Fig. Leg.	5,6<br><br>(2-6)	Animals per group<br>(slices per animal)	Fig. Leg<br><br>(Methods)	Error bars are mean +/- SEM	Results, "Statistics"	p=0.0146	-	t(9)=3.016	-
2J	2Way ANOVA<br><br>Fisher's LSD	Fig. Leg.	5,6<br><br>(5-20)	Animals per group<br>(slices per animal)	Fig. Leg<br><br>(Methods)	Error bars are mean +/- SEM	Results, "Statistics"	**Condition:** p=0.0082<br>**Region:** p<0.0001<br>**Interaction:** p=0.4722	-	F(1,72)=7.386<br>F(7,72)=13.51<br>F(7,72)=0.9526	-
4J	Kruskal-Wallis test<br><br>Dunn's post-hoc<br><br>Failed normality	Fig. Leg.	11-15<br><br>(5-16)	Animals per group<br><br>(slices per animal)	Fig. Leg.	Error bars are mean +/- SEM	Results, "Statistics"	**Condition:** p<0.0001	-	H=50.11	-
4L	2Way ANOVA<br><br>Tukey's post-hoc	Fig. Leg.	11-15<br><br>(5-16)	Animals per group<br>(slices per animal)	Fig. Leg.	Error bars are mean +/- SEM	Results, "Statistics"	**Circuit:** p=0.7473<br>**Subregion:** p<0.0001<br><br>**Interaction:** p<0.0001	-	F(5,69)=0.5374<br>F(1.671,115.3)= 63.67<br>F(10,138)=39.3 3	-

	Test Used		n			Descriptive stats		p-value		DEGREES OF FREEDOM & F/t/z/R/ETC VALUE	
Figure Number	Which test?	Section & paragraph #	Exact value	Defined	Section & paragraph #	Reported	Section & paragraph #	Exact value	Section & paragraph #	Value	Section & Paragraph #
5C	Mann-Whitney test<br><br>Failed normality	Fig. Leg.	4, 8<br><br>(9-16)	Animals per group (slices/animal)	Fig. Leg.	Error bars are mean +/- SEM	Results, "Statistics"	p=0.0020	-	-	-
5D	Unpaired t-test	Fig. Leg	5, 6<br><br>(8-12)	Animals per group (slices/animal)	Fig. Leg.	Error bars are mean +/- SEM	Results, "Statistics"	p<0.0001	-	t(9)=10.28	-
5E	Mann-Whitney test<br><br>Failed normality	Fig. Leg.	5, 6<br><br>(5-15)	Animals per group (slices/animal)	Fig. Leg.	Error bars are mean +/- SEM	Results, "Statistics"	p=0.0043	-	-	-
5F	Unpaired t-test	Fig. Leg.	6,7<br><br>(7-15)	Animals per group (slices/animal)	Fig. Leg.	Error bars are mean +/- SEM	Results, "Statistics"	p=0.0011	-	t(11)=4.393	-
5G	Mann-Whitney test<br><br>Failed normality	Fig. Leg.	7,8<br><br>(6-15)	Animals per group (slices/animal)	Fig. Leg.	Error bars are mean +/- SEM	Results, "Statistics"	p=0.0012	-	-	-
5H	Unpaired t-test	Fig. Leg.	6,5<br><br>(7-13)	Animals per group (slices/animal)	Fig. Leg.	Error bars are mean +/- SEM	Results, "Statistics"	p=0.0339	-	t(9)=2.499	-
5I	Kruskal-Wallis test<br><br>Dunn's post-hoc<br><br>Failed normality	Fig. Leg.	4,5,5, 7,7,6	Animals per group	Fig. Leg.	Error bars are mean +/- SEM	Results, "Statistics"	p<0.0001	-	(H)=28.03	-
5M	RM one-way ANOVA<br><br>Tukey's post-hoc	Fig. Leg.	4	Animals	Fig. Leg.	Error bars are mean +/- SEM	Results, "Statistics"	p=0.0117		F(1.057,3.172) =27.20	-
5N	RM one-way ANOVA<br><br>Tukey's post-hoc	Fig. Leg.	5	Animals	Fig. Leg.	Error bars are mean +/- SEM	Results, "Statistics"	p=0.0005		F(1.468, 5.870) =41.28	-
5O	RM one-way ANOVA<br><br>Tukey's post-hoc	Fig. Leg.	6	Animals	Fig. Leg.	Error bars are mean +/- SEM	Results, "Statistics"	p=0.0019		F(1.307,6.537) =22.23	-
5P	RM one-way ANOVA<br><br>Tukey's post-hoc	Fig. Leg.	7	Animals	Fig. Leg.	Error bars are mean +/- SEM	Results, "Statistics"	p=0.0068		F(1.093,6.556) =14.58	-
5Q	RM one-way ANOVA<br><br>Tukey's post-hoc	Fig. Leg.	7	Animals	Fig. Leg.	Error bars are mean +/- SEM	Results, "Statistics"	p=0.3641	-	F(1.753,10.52)	-
5R	RM one-way ANOVA<br><br>Tukey's post-hoc	Fig. Leg.	5	Animals	Fig. Leg.	Error bars are mean +/- SEM	Results, "Statistics"	p=0.0040		F(1.680,6.720)	-
6E	Unpaired t-test	Fig. Leg.	10,13	Animals per group	Fig. Leg.	Error bars are mean +/- SEM	Results, "Statistics"	p=0.1592	-	t(21)=1.459	-
6F	Mann-Whitney test<br><br>Failed normality	Fig. Leg.	10,13	Animals per group	Fig. Leg.	Error bars are mean +/- SEM	Results, "Statistics"	p=0.3353	-	-	-
6G	Unpaired t-test	Fig. Leg.	10,13	Animals per group	Fig. Leg.	Error bars are mean +/- SEM	Results, "Statistics"	p=0.0099	-	t(21)=2.834	-
6H	Mixed effects analysis<br><br>Fisher's LSD	Fig. Leg.	10, 9, 14	Animals per group	Fig. Leg.	Error bars are mean +/- SEM	Results, "Statistics"	**Condition:** p=0.9319	-	F(2,59)=0.0706	-
6I	Mixed effects analysis<br><br>Fisher's LSD	Fig. Leg.	9,10	Animals per group	Fig. Leg.	Error bars are mean +/- SEM	Results, "Statistics"	**Condition:** p=0.0103	-	F(1,33)=7.411	-
6J	2Way ANOVA<br><br>Fisher's LSD	Fig. Leg.	8,6	Animals per group	Fig. Leg.	Error bars are mean +/- SEM	Results, "Statistics"	**Condition:** p=0.5875	-	F(1,12)=0.3107	-
6K	2Way ANOVA<br><br>Fisher's LSD	Fig. Leg.	6,8	Animals per group	Fig. Leg.	Error bars are mean +/- SEM	Results, "Statistics"	**Condition:** p=0.6693	-	F(1,12)=0.1917	-
6L	2Way ANOVA<br><br>Fisher's LSD	Fig. Leg.	7,8	Animals per group	Fig. Leg.	Error bars are mean +/- SEM	Results, "Statistics"	**Condition:** p=0.5078	-	F(1,12)=0.4661	-

Table S3. Fisher's LSD, Fig. 1G

Comparison (VEH vs CNO)		Adjusted P Value
t=0	ns	0.8187
t=30	***	0.0001

Table S4. Fisher's LSD, Fig. 2F

Comparison (mCherry vs ChR2)		Adjusted P Value
t=30	ns	0.3560
t=60	ns	0.0597
t=90	*	0.0214

Table S5. Fisher's LSD, Fig. 2J

Comparison (mCherry vs ChR2)		Adjusted P Value
AcbC	ns	0.1842
AcbS	ns	0.8369
PrL	*	0.0147
LH	ns	0.6224
CeA	ns	0.5097
dmBST	*	0.0282
aBST	ns	0.6231
VH	ns	0.8705

Table S6. Dunn's post-hoc, Fig. 4J

Comparison (mCherry vs ChR2)		Adjusted P Value
BLA-PrL vs. BLA-NAc	**	0.0022
BLA-PrL vs. BLA-BST	ns	0.6364
BLA-PrL vs. BLA-CeA	*	0.0135
BLA-PrL vs. BLA-LH	ns	>0.9999
BLA-PrL vs. BLA-VH	ns	>0.9999
BLA-NAc vs. BLA-BST	ns	>0.9999
BLA-NAc vs. BLA-CeA	ns	>0.9999
BLA-NAc vs. BLA-LH	****	<0.0001
BLA-NAc vs. BLA-VH	****	<0.0001
BLA-BST vs. BLA-CeA	ns	>0.9999
BLA-BST vs. BLA-LH	**	0.0091
BLA-BST vs. BLA-VH	*	0.0474
BLA-CeA vs. BLA-LH	****	<0.0001
BLA-CeA vs. BLA-VH	***	0.0003
BLA-LH vs. BLA-VH	ns	>0.9999

Table S7. Tukey's post-hoc, Fig. 4L

Comparison (mCherry vs ChR2)		Adjusted P Value
PrL		
mBA vs. LA	****	<0.0001
mBA vs. LBA	****	<0.0001
LA vs. LBA	ns	0.2340
NAc		
mBA vs. LA	****	<0.0001
mBA vs. LBA	****	<0.0001
LA vs. LBA	ns	0.8019
BST		
mBA vs. LA	****	<0.0001
mBA vs. LBA	****	<0.0001
LA vs. LBA	ns	0.2863
CeA		
mBA vs. LA	***	0.0005
mBA vs. LBA	****	<0.0001
LA vs. LBA	ns	0.8884
LH		
mBA vs. LA	ns	0.8489
mBA vs. LBA	****	<0.0001
LA vs. LBA	**	0.0026
VH		
mBA vs. LA	ns	0.7111
mBA vs. LBA	**	0.0029
LA vs. LBA	ns	0.1218

Table S8. Dunn's post-hoc, Fig. 5I

Comparison (mCherry vs ChR2)		Adjusted P Value
BLA-PrL vs. BLA-NAc	ns	0.0637
BLA-PrL vs. BLA-BST	ns	>0.9999
BLA-PrL vs. BLA-CeA	ns	0.7964
BLA-PrL vs. BLA-LH	ns	>0.9999
BLA-PrL vs. BLA-VH	ns	>0.9999
BLA-NAc vs. BLA-BST	ns	>0.9999
BLA-NAc vs. BLA-CeA	ns	>0.9999
BLA-NAc vs. BLA-LH	***	0.0001
BLA-NAc vs. BLA-VH	**	0.0082
BLA-BST vs. BLA-CeA	ns	>0.9999
BLA-BST vs. BLA-LH	ns	0.0813
BLA-BST vs. BLA-VH	ns	0.9310
BLA-CeA vs. BLA-LH	**	0.0062
BLA-CeA vs. BLA-VH	ns	0.1890
BLA-LH vs. BLA-VH	ns	>0.9999

Table S9. Tukey's multiple comparisons, Fig. 5M

Comparison		Adjusted P Value
LA vs. mBA	*	0.0358
LA vs. LBA	ns	0.7813
mBA vs. LBA	*	0.0180

Table S10. Tukey's multiple comparisons, Fig. 5N

Comparison		Adjusted P Value
LA vs. mBA	**	0.0088
LA vs. LBA	ns	0.7179
mBA vs. LBA	***	0.0009

Table S11. Tukey's multiple comparisons, Fig. 5O

Comparison		Adjusted P Value
LA vs. mBA	*	0.0250
LA vs. LBA	ns	0.4658
mBA vs. LBA	***	0.0010

Table S12. Tukey's multiple comparisons, Fig. 5P

Comparison		Adjusted P Value
LA vs. mBA	*	0.0180
LA vs. LBA	*	0.0202
mBA vs. LBA	ns	0.6866

Table S13. Tukey's multiple comparisons, Fig. 5Q

Comparison		Adjusted P Value
LA vs. mBA	ns	0.5522
LA vs. LBA	ns	0.9568
mBA vs. LBA	ns	0.4837

Table S14. Tukey's multiple comparisons, Fig. 5R

Comparison		Adjusted P Value
LA vs. mBA	*	0.0441
LA vs. LBA	ns	0.7215
mBA vs. LBA	*	0.0151

Table S15. Fisher's LSD, Fig. 6H

**Comparison**		**Adjusted P Value**
t=0		
hM4Di/VEH vs. mCherry/CNO	ns	0.9166
hM4Di/VEH vs. hM4Di/CNO	ns	0.9324
mCherry/CNO vs. hM4Di/CNO	ns	0.9755
t=30		
hM4Di/VEH vs. mCherry/CNO	ns	0.8652
hM4Di/VEH vs. hM4Di/CNO	ns	0.6530
mCherry/CNO vs. hM4Di/CNO	ns	0.7999

Table S16. Fisher's LSD, Fig. 6I

**Comparison (VEH vs. CNO)**		**Adjusted P Value**
t=0	ns	0.4653
t=30	**	0.0035

Table S17. Fisher's LSD, Fig. 6J

**Comparison (VEH vs. CNO)**		**Adjusted P Value**
t=0	ns	0.7333
t=30	ns	0.2936

Table S18. Fisher's LSD, Fig. 6K

**Comparison (VEH vs. CNO)**		**Adjusted P Value**
t=0	ns	0.7901
t=30	ns	0.6793

Table S19. Fisher's LSD, Fig. 6L

**Comparison (VEH vs. CNO)**		**Adjusted P Value**
t=0	ns	0.5182
t=30	ns	0.1102
